# Cancer stem cells synthesize proline to attenuate oxidative stress

**DOI:** 10.1172/JCI200775

**Published:** 2026-06-01

**Authors:** Weichi Wu, Po Zhang, Donghai Wang, Xujia Wu, Qiulian Wu, Daqi Li, Tengfei Huang, Rui Wang, Huan Li, Hailong Mi, Suchet Taori, Fanen Yuan, Tingting Duan, Zhiye Chen, Huairui Yuan, Jeremy N. Rich

**Affiliations:** 1 Lineberger Comprehensive Cancer Center, University of North Carolina, Chapel Hill, North Carolina, USA.; 2 UPMC Hillman Cancer Center, Pittsburgh, Pennsylvania, USA.; 3 Department of Medicine, and; 4 Department of Neurology, University of Pittsburgh, Pittsburgh, Pennsylvania, USA.; 5 Department of Neurology, University of North Carolina, Chapel Hill, North Carolina, USA.

**Keywords:** Cell biology, Metabolism, Oncology, Amino acid metabolism, Brain cancer, Human stem cells

## Abstract

Cancers reprogram their metabolism to provide anabolic needs without driving excessive oxidative stress. Attention has focused on glucose metabolism, yet amino acid synthesis and degradation also promote tumor cell states and growth. Here, we assessed amino acids that maintain cancer stem cells in glioblastoma and found increased proline levels relative to differentiated tumor progeny through increased proline synthesis. Cancer stem cells preferentially expressed the signaling molecule FAM3C induced by the stem cell transcription factor SOX2 to drive expression of proline synthesis enzymes. FAM3C classically mediated cellular responses as a secreted protein but gained intracellular functions in cancer stem cells through binding the histone reader spindlin 1 (SPIN1), thereby preventing its lysosomal degradation, assisting its nuclear localization, and promoting epigenetic regulation of proline synthesis. Proline synthesis depleted ROS, and genetic targeting of FAM3C attenuated ROS scavenging, whereas SPIN1 OE restored ROS levels. Molecular docking identified tucatinib as a brain-penetrant pharmacologic disruptor of FAM3C-SPIN1 interactions, promoting SPIN1 degradation and reducing intracellular proline levels. Thus, cancer stem cells induced a favorable metabolic state through proline synthesis and ROS depletion, revealing potential therapeutic dependencies.

## Abstract 2

Cancer stem cells drive brain tumor growth by producing proline to neutralize stress; <span><span>Tucatinib emerges as a potential drug to disrupt this metabolic shield.</span></span> <span><source-footnote _nghost-ng-c350547919=”” ng-version=”0.0.0-PLACEHOLDER”></source-footnote><source-footnote _nghost-ng-c350547919=”” ng-version=”0.0.0-PLACEHOLDER”></source-footnote><source-footnote _nghost-ng-c350547919=”” ng-version=”0.0.0-PLACEHOLDER”></source-footnote></span>

## Introduction

Glioblastoma (GBM) (WHO grade 4 glioma) is the most prevalent and lethal primary intrinsic brain tumor, characterized by rapid progression and resistance to conventional treatment. Despite multimodal therapy consisting of surgical resection, concurrent chemoradiotherapy, and adjuvant chemotherapy with temozolomide (TMZ), median patient survival remains limited to less than 2 years, even in patients with favorable characteristics (1, 2). GBM is originally designated as GBM multiforme, befitting its remarkable morphologic heterogeneity, which has recently been validated in spatial and temporal heterogeneity of genomic, epigenomic, proteomic, and metabolomic landscapes (3). Diversity within the tumor cell compartment has been described with multiple models, including stochastic and hierarchical models. Atop cellular hierarchies, self-renewing, stem-like tumor cells have been designated as cancer stem cells. GBM stem cells (GSCs) are a resilient tumor cell population that contributes to radiotherapy and chemotherapy resistance (4–6). While the precise influence of GSCs remains a subject of investigation, unraveling their molecular regulatory mechanisms could pave the way for innovative treatment strategies, ultimately improving patient outcomes in GBM therapy.

Tumor metabolism has undergone a renaissance, having once been considered simply a byproduct of oncogenesis, but the discovery of driver mutations in metabolic enzymes, including isocitrate dehydrogenases 1 and 2 (IDH1/2) in gliomas, has demonstrated that metabolism can drive tumor initiation and progression (7–9). Glucose metabolism has been a dominant focus of many cancer metabolism studies, but GBMs also take up amino acids to promote tumor growth through multiple molecular mechanisms (10). We and others have reported that different amino acids display distinct downstream regulatory mechanisms promoting brain tumor growth. Methionine fuels accumulation of methyl donor precursors, leading to dysregulation of DNA methylation and expression of oncogenes (11). Lysine accumulates preferentially in GSCs, in which it undergoes degradation to generate an intermediate, crotonyl-CoA, which acts as an epigenetic mark to repress the expression of endogenous retroviral elements (ERVs) (12). Threonine serves as a cofactor for tRNA regulators YRDC and the KEOPS complex to facilitate codon-specific protein translation (13). Collectively, these studies and others suggest that amino acids not only serve anabolic processes but also induce the dysregulation of distinct oncogenic pathways.

Among amino acids, proline is unique because of its cyclic structure (technically, it is a secondary amine), in which the side chain is bonded to both the nitrogen and the α-carbon of the amino acid backbone, creating a ring. Other amino acids have a free amino group attached to the α-carbon. This unique structure gives proline distinct properties that influence protein structure and function, including serving critical roles in molecular recognition sequences. Proline metabolism plays crucial roles in cellular energy production and protein and nucleotide synthesis and maintains redox homeostasis (14). Studies across diverse cancer cell lines indicate that proline deprivation or inhibition of enzymes involved in its biosynthesis impairs clonal expansion and tumorigenic potential, highlighting its importance in sustaining malignant growth (15, 16).

Proline synthesis initiates within the mitochondria with the precursor glutamate, which primarily originates from glutamine (17). The process starts with transformation into pyrroline-5-carboxylate (P5C), a reaction catalyzed by P5C synthase (P5CS) (ALDH18A1) (17, 18). Next, mitochondrial P5C reductases (PYCR1 and PYCR2) facilitate the reduction of P5C, ultimately producing proline (17, 19). Proline biosynthetic enzymes (i.e., P5CS, PYCR1, and PYCR2) are expressed in GBM (20), suggesting that proline may contribute to GBM growth.

Here, we found that GSCs contained elevated levels of proline and FAM3C upstream of proline biosynthesis and that proline synthesis was linked to ROS scavenging. Drug screening identified tucatinib targeting of FAM3C function as a means to inhibit proline biosynthesis and proliferation in GSCs, revealing a potential therapeutic strategy for GBM treatment.

## Results

### GSCs upregulate proline biosynthesis and proline-related genes.

**Figure 1:**
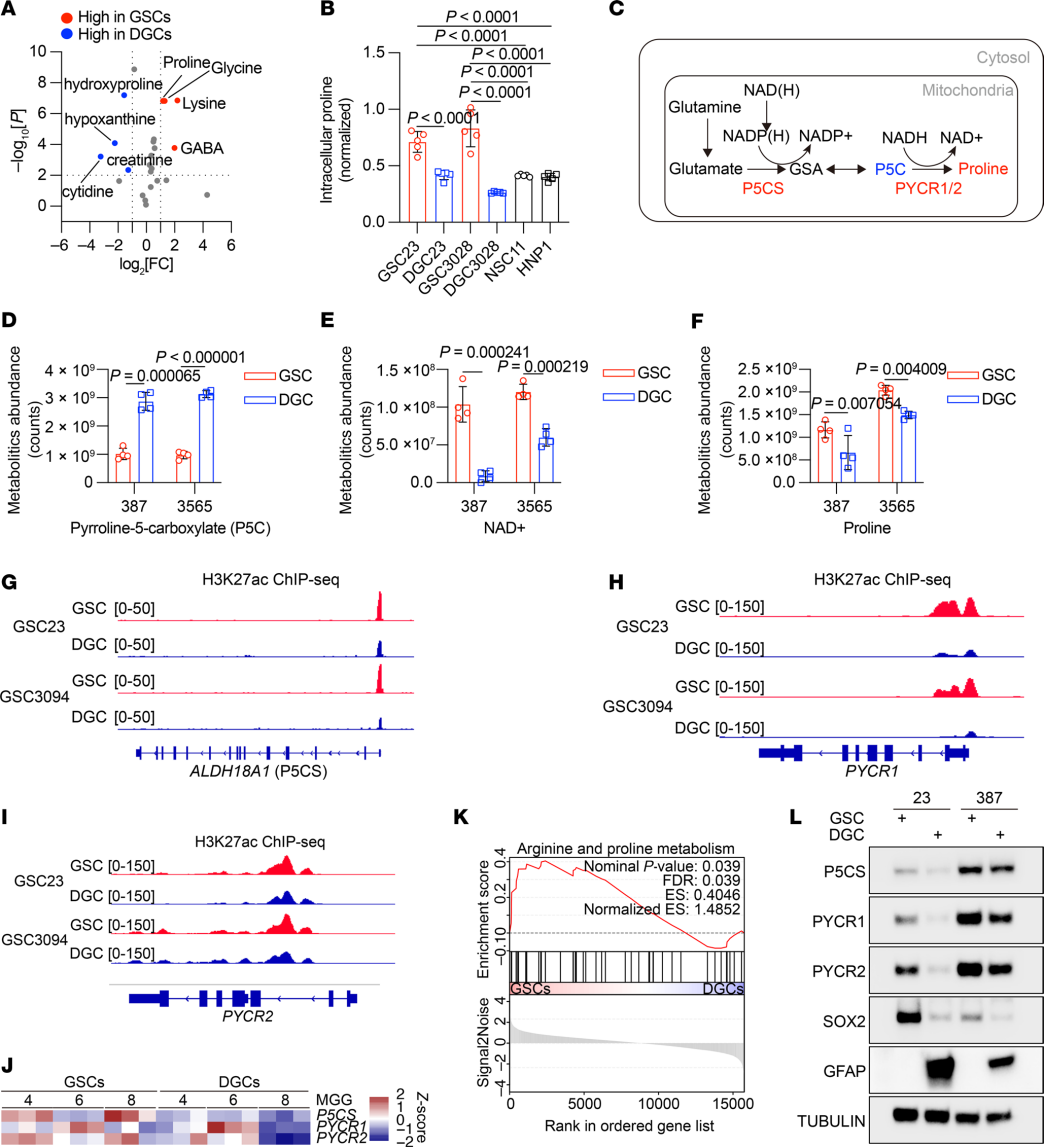
GSCs upregulate proline biosynthesis and proline-related genes. (**A**) Summary of intracellular amino acid levels across GSCs and DGCs from 5 biological replicates (12). The fold changes (FCs) in amino acid levels relative to the average of GSCs are displayed. Statistical analysis was determined by 2-tailed, unpaired *t* test. (**B**) Summary of intracellular proline levels across GSCs and NSCs from 5 biological replicates (12). Data are presented as the mean ± SD. Statistical significance was determined by 1-way ANOVA followed by a multiple-comparison test. (**C**) Schematic of the proline biosynthesis pathway in mitochondria. (**D**–**F**) P5C (**D**), NAD^+^ (**E**), and proline (**F**) detected by LC-MS in 2 CD133^+^ GSC lines and paired CD133^–^ DGCs (*n* = 4 independent experiments) (21). Data are presented as the mean ± SD. Statistical significance was determined by 2-tailed, unpaired *t* test. (**G**–**I**) H3K27ac ChIP-seq tracks at ALDH18A1 (P5CS) (**G**), PYCR1 (**H**), and PYCR2 (**I**) gene loci (GSE129438). (**J**) Summary of gene expression in matched GSCs and DGCs (GSE54791). (**K**) GSEA of arginine and proline metabolism among DEGs from matched GSCs and DGCs. Normalized enrichment score (ES) and nominal *P* values are shown. (**L**) Immunoblot analysis of matched GSCs and DGCs.

We previously reported that GSCs contain more lysine and other selected amino acids than do differentiated GBM cells (DGCs) (12, 21). As we and others have found that amino acids have distinct regulatory effects on GSCs, we interrogated our metabolic data to determine if proline was likewise differentially regulated. GSCs showed elevation of proline, lysine, glycine, and GABA compared with DGCs (Figure 1A). GSCs also contained elevated proline levels compared with human neural stem cells (NSCs) (Figure 1B). We hypothesized that proline levels were elevated in GSCs due to increased proline synthesis in mitochondria, which starts with glutamate largely derived from glutamine (Figure 1C) (17). P5CS converts glutamate into P5C (17, 18), which is then reduced by mitochondrial P5C reductases (PYCR1 and PYCR2) to generate proline (17, 19). We performed targeted metabolomics of P5C, an intermediate product in the synthesis of proline, NAD^+^, a byproduct in the final synthesis of proline, and proline itself in matched pairs of GSCs and DGCs. Consistent with increased activity of PYCR1/2 in GSCs, P5C levels were lower than in DGCs (Figure 1D), whereas NAD^+^ and proline levels were higher compared with levels in DGCs (Figure 1, E and F).

To assess the regulation of the essential proline biosynthetic enzymes, we interrogated histone 3 lysine 27 acetyl (H3K27ac) ChIP-seq from 2 pairs of matched GSCs and DGCs (22). Consistent with increased transcription of *ALDH18A1* (encodes the P5CS protein), *PYCR1*, and *PYCR2*, H3K27ac deposition was greater on each gene in GSCs compared with DGCs (Figure 1, G–I). RNA-seq of 3 pairs of matched GSCs and DGCs confirmed increased transcripts of these 3 genes were higher in GSCs compared with DGCs (Figure 1J). Gene set enrichment analysis (GSEA) indicated enrichment of arginine and proline metabolism in GSCs (Figure 1K). To confirm high protein levels of P5CS, PYCR1, and PYCR2 in GSCs, we induced GSC differentiation into DGCs. We validated the differentiation by downregulation of the GSC marker SOX2 and upregulation of the lineage marker GFAP, measured by immunoblot (Figure 1L). GSCs indeed exhibited higher protein levels of key proline synthesis enzymes compared with DGCs (Figure 1L).

### FAM3C regulates proline biosynthesis in GSCs.

**Figure 2:**
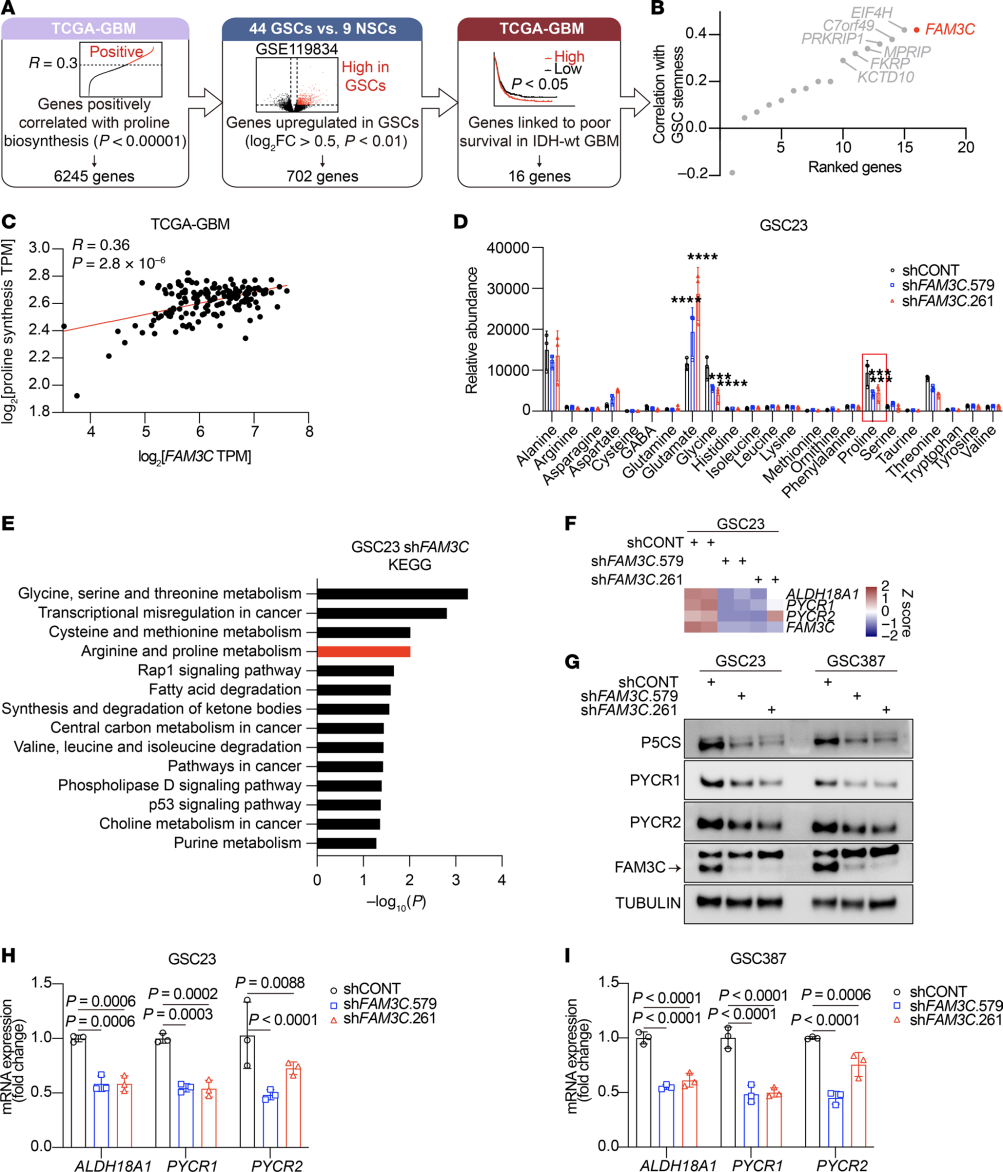
FAM3C regulates proline biosynthesis in GSCs. (**A**) Diagram depicting the screening strategy to identify potential regulators of proline synthesis in GSCs. (**B**) Rank of 16 genes identified in **A** ranked according to their correlation with the GSC stemness index in TCGA-GBM. The top 7 ranked genes are labeled. (**C**) Pearson’s correlation of FAM3C expression and proline synthesis in RNA-seq data of TCGA_GBM (*n* = 150). The red line shows linear regression. (**D**) Changes of cellular metabolite levels measured by LC-MS in GSC23 cells with shCONT, sh*FAM3C*.579, or sh*FAM3C*.261 for 48 hours (*n* = 3 independent experiments). ***P < 0.001 and ****P < 0.0001, by 2-tailed, unpaired *t* test. (**E**) KEGG pathway analysis of downregulated genes after *FAM3C* KD in GSC23 cells, ranked by *P* values. (**F**) Heatmap summarizing the differential transcript expression of *ALDH18A1* (P5CS), *PYCR1*, and *PYCR2* between control and *FAM3C*-KD GSCs. (**G**) Immunoblot analysis of key enzymes in proline synthesis in GSCs with or without *FAM3C* KD. (**H** and **I**) qRT-PCR analysis of key enzymes in proline synthesis in GSC23 (**H**) and GSC387 (**I**) with or without *FAM3C* KD (*n* = 3 independent experiments). Data are presented as the mean ± SD. Statistical analysis was performed using a 1-way ANOVA followed by a multiple-comparison test.

Elevated proline and biosynthetic enzyme levels in GSCs prompted us to investigate potential regulators of proline synthesis in GSCs. Therefore, we followed a sequential discovery approach. First, we selected genes positively correlated with proline biosynthesis in The Cancer Genome Atlas (TCGA) GBM dataset, of which there were 6,245 (Figure 2A). Next, we interrogated our previously published gene expression dataset on 44 patient-derived GSCs and 9 human NSC lines (23) to reveal that 702 genes from this gene set were preferentially expressed in GSCs. Further refinement revealed that 16 of these genes negatively correlated with patient survival in isocitrate dehydrogenase WT (IDH-WT) GBMs in TCGA, among which the gene encoding the secreted protein FAM3 metabolism-regulating signaling molecule C (*FAM3C*) exhibited the strongest correlation with GSC stemness (24) (Figure 2B). *FAM3C* positively correlated with a gene signature of proline synthesis (Figure 2C).

To confirm the functional connection between FAM3C and proline levels, patient-derived GSCs were transduced with 1 of 2 nonoverlapping shRNAs targeting *FAM3C* (designated as shFAM3C.261 and shFAM3c.579) or a nontargeting control shRNA (shCONT), followed by mass spectrometry (MS) analysis of free amino acid levels. FAM3C (KD) reduced proline levels, accompanied by an increase in glutamate levels (Figure 2D). Silencing *FAM3C* in GSCs, followed by RNA-seq and Kyoto Encyclopedia of Genes and Genomes (KEGG) analyses, showed reduced arginine and proline metabolism genes (Figure 2E), which included *ALDH18A1*, *PYCR1*, and *PYCR2* (Figure 2F). Protein levels of these 3 key enzymes in proline synthesis were also reduced following KD of FAM3C (Figure 2G). Quantitative reverse transcription PCR (qRT-PCR) analysis confirmed that FAM3C regulated *ALDH18A1*, *PYCR1*, and *PYCR2* transcription (Figure 2, H and I).

### SOX2 drives FAM3C expression in GSCs.

**Figure 3:**
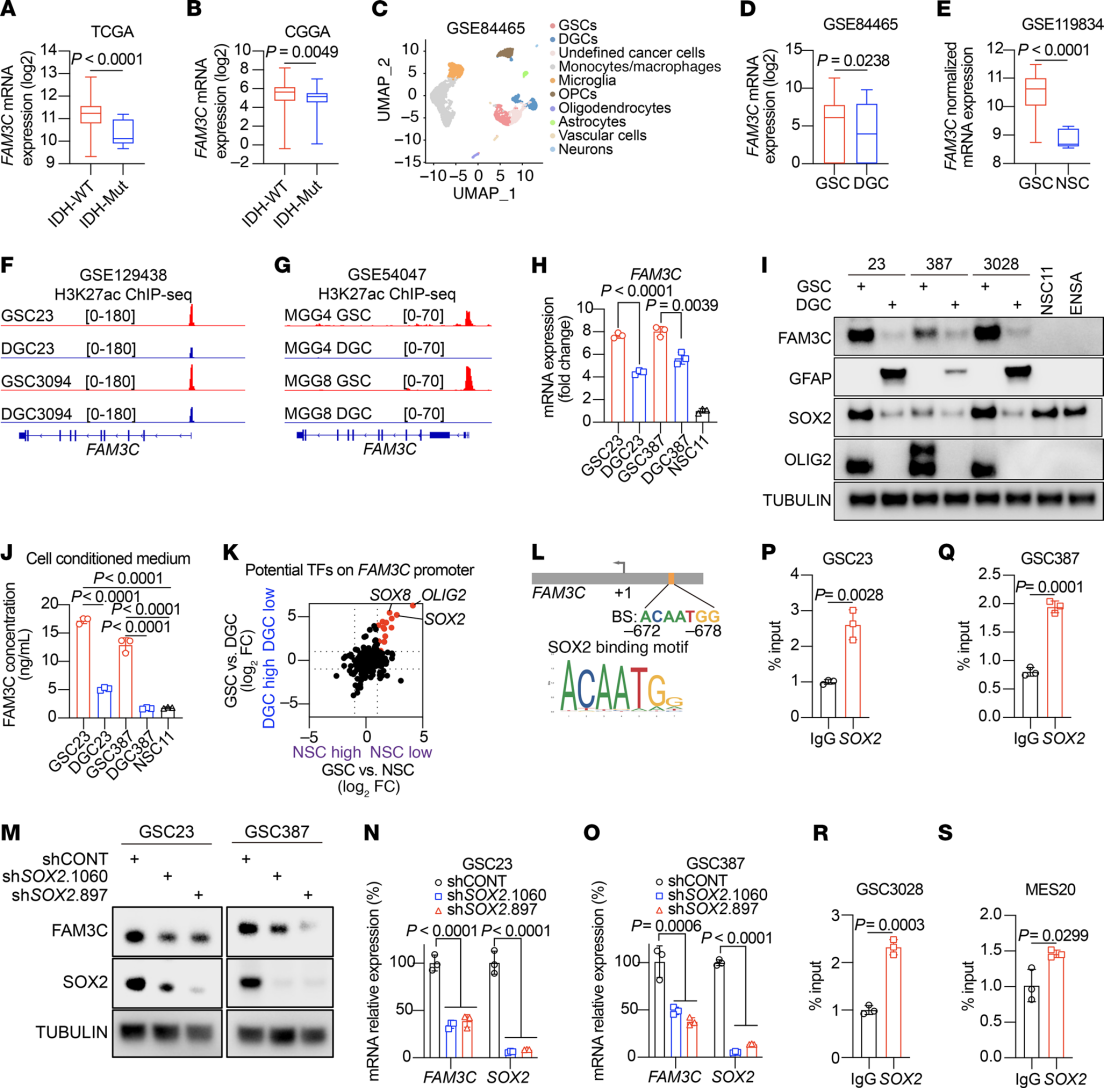
SOX2 drives FAM3C expression in GSCs. (**A** and **B**) FAM3C expression in IDH-WT (142 samples in TCGA and 287 samples in CGGA) and IDH-Mut (8 samples in TCGA and 324 samples in CGGA) glioma samples in TCGA (**A**) and the CGGA (**B**). (**C**) 2D uniform manifold approximation and projection (UMAP) representation of 3,589 single cells in GSE84465. (**D**) Expression of *FAM3C* in 449 GSCs and 418 DGCs as determined by the scRNA-seq analysis shown in **C**. (**E**) Expression of *FAM3C* in GSCs (*n* = 41 samples) and NSCs (*n* = 5 samples) (GSE119834). (**F** and **G**) H3K27ac ChIP-seq tracks at *FAM3C* gene loci in GSCs and DGCs: GSE129438 (**F**) and GSE54047 (**G**). (**H**) qRT-PCR analysis of *FAM3C* in GSCs, DGCs, and NSCs (*n* = 3 independent experiments). (**I**) Immunoblots of GSCs, DGCs, and NSCs. (**J**) FAM3C levels measured by ELISA (abx387273 kit, Abbexa) in neurobasal medium was used to culture GSCs, DGCs, or NSCs for 48 hours (*n* = 3 independent experiments). (**K**) Comparison of the expression of TFs potentially associated with *FAM3C* in RNA-seq data for GSCs versus DGCs (GSE54791) and GSCs versus NSCs (GSE119834). Dashed lines show the cutoff (|log_2_FC|>1). Red dots indicate TFs enriched in GSCs. (**L**) Graphic illustration of the predicted SOX2 BS on the *FAM3C* promoter. (**M**) Immunoblots of GSCs with or without SOX2 KD. (**N** and **O**) qRT-PCR analysis of GSC23 (**N**) and GSC387 (**O**) with or without *SOX2* KD (*n* = 3 independent experiments). (**P**–**S**) SOX2 ChIP-qPCR analysis in GSCs: GSC23 (**P**), GSC387 (**Q**), GSC3028 (**R**), and MES20 (**S**) detecting the indicated BS in **L** (*n* = 3 independent experiments). In **A**, **B** and **D**, and **E**, Boxes represent data within the 25th–75th percentiles. Whiskers depict the range of all data points. Horizontal lines within boxes represent mean values. In **H**, **J**, and **N**–**S**, data are presented as the mean ± SD. Statistical significance was determined by 2-tailed, unpaired *t* test (**A**, **B**, **D**, **E**, **H**, and **P**–**S**) and 1-way ANOVA followed by a multiple-comparison test (**J** and **N** and **O**).

FAM3C is also referred to as IL-like epithelial-mesenchymal transition (EMT) inducer (ILEI) (25). This recently characterized protein plays roles in various biological functions, including hepatic glucose and lipid metabolism, osteogenic differentiation, and EMT (26, 27). Network analysis revealed elevated FAM3C expression in GBM (28). We found higher FAM3C expression levels in IDH-WT GBM than in IDH-mutant (IDH-Mut) GBM (Figure 3, A and B). To measure specific FAM3C expression levels in GSCs, we analyzed in silico single-cell data from patient-derived GBMs segregated between cell types, including GSCs and DGCs (Figure 3C) (29). *FAM3C* expression was higher in GSCs compared with DGCs (Figure 3D). We analyzed our previously reported RNA-seq dataset comprising GSCs and NSCs, revealing FAM3C expression in GSCs was markedly elevated compared with NSCs (Figure 3E) (23). ChIP-seq analysis of H3K27ac revealed that FAM3C had higher transcriptional activity in GSCs than in DGCs or NSCs (Figure 3, F and G, and Supplemental Figure 1A; supplemental material available online with this article; https://doi.org/10.1172/jci.200775) (22, 23, 30). We independently validated these results at both the protein and mRNA levels in paired GSC and DGC samples, as well as in NSCs, using immunoblotting and reverse transcription PCR (RT-PCR), respectively (Figure 3, H and I). FAM3C functions as a cytokine-like secreted protein (31), so we measured its secretion by ELISA, confirming that GSCs secreted higher levels of FAM3C than DGCs or NSCs (Figure 3J and Supplemental Figure 1B). Based on immunohistochemical staining data from the Human Protein Atlas, we observed that FAM3C expression was higher in high-grade glioma (HGG) compared with low-grade glioma (LGG) (Supplemental Figure 1C).

To understand the upstream regulation of FAM3C specific to GSCs, we correlated over 500 potential transcription factors (TFs) with regulation based on the FAM3C promoter in GSCs relative to DGCs and NSCs (Figure 3K). The top 3 TFs were OLIG2, SOX2, and SOX8 (Figure 3K) (23, 30). Among these TFs in GBM datasets in both TCGA and the Chinese Glioma Genome Atlas (CGGA), SOX2 was the most tightly correlated with FAM3C (Supplemental Figure 1D). A potential SOX2 binding site (BS) was predicted 672–678 bp upstream of the predicted FAM3C start site (Figure 3L). SOX2 KD decreased FAM3C protein and transcription levels in GSCs (Figure 3, M–O). The potential SOX2 BS was verified by ChIP followed by qPCR (ChIP-qPCR) analysis in 4 GSCs (Figure 3, P–S). SOX2 ChIP-seq analysis in 2 GSC lines (MES20 and GSC3028) revealed a peak at the FAM3C promoter, indicating that SOX2 directly bound to the FAM3C locus (Supplemental Figure 1E). To address why NSCs express SOX2 but do not highly express FAM3C, we first confirmed that SOX2 remained a functional regulator of FAM3C in NSCs; KD of SOX2 also decreased FAM3C levels in 2 NSCs (Supplemental Figure 1F). By analyzing public ATAC-seq data across GSCs, DGCs, and NSCs (32–34), we observed the FAM3C promoter region had high chromatin accessibility in GSCs (e.g., GSCs 456, 387, and 4121) (Supplemental Figure 1G). In contrast, ATAC-seq tracks for multiple NSCs (including WA09-derived NSCs, GCGR-NS13, and GCGR-NS19) revealed lower signals at the FAM3C promoter. This epigenetic restriction likely limits the transcriptional efficiency of SOX2 on the FAM3C gene in normal NSCs. Collectively, SOX2, which has been strongly connected to GSC maintenance, promotes FAM3C expression in GSCs.

### FAM3C regulates SPIN1 stability and nuclear translocation to fuel proline biosynthesis.

**Figure 4:**
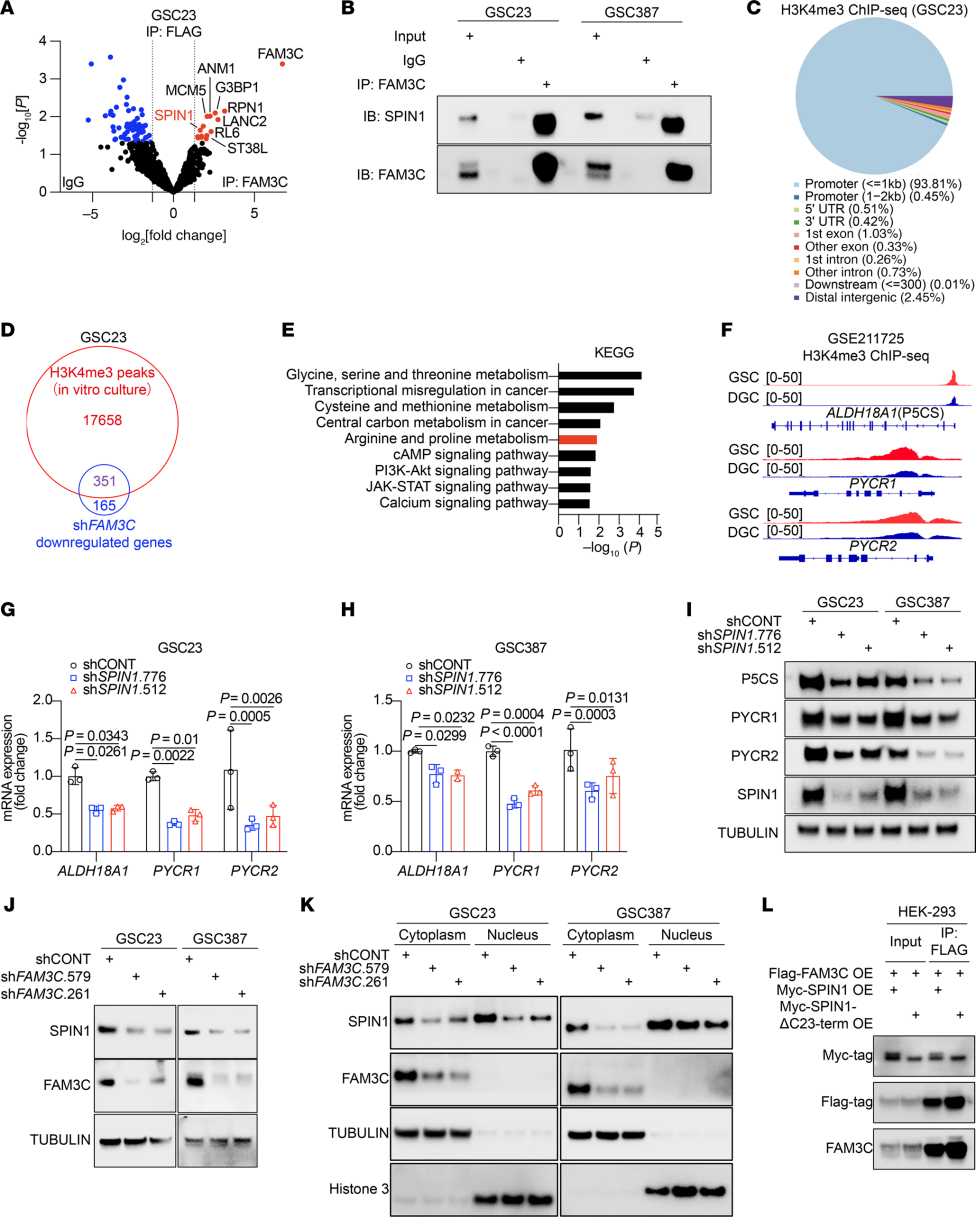
FAM3C interacts with SPIN1. (**A**) Volcano plot showing proteins that interact with FAM3C in GSCs (GSC23) through proteomics data (FAM3C IP vs. IgG) (cutoff, |log_2_ (FC)| > 0.5, *P* < 0.05) (*n* = 2 biologically independent samples). Red dots indicate proteins enriched in GSCs and interacting with the FAM3C protein. (**B**) Immunoblot of FAM3C and SPIN1 protein immunoprecipitates in GSCs (GSC23 and GSC387). (**C**) Pie chart showing the genomic distribution of H3K4me3 peaks in GSC23 cells by H3K4me3 ChIP-seq (GSE185954). (**D**) Venn diagram showing 351 genes marked by H3K4me3 peaks that were downregulated following *FAM3C* KD. (**E**) KEGG pathway analysis of genes identified in **D** ranked by *P* value. (**F**) H3K4me3 ChIP-seq tracks at ALDH18A1 (P5CS), PYCR1, and PYCR2 gene loci (GSE211725). (**G** and **H**) qRT-PCR analysis of key enzymes in proline synthesis in GSC23 (**G**) and GSC387 (**H**) GSCS with or without *SPIN1* KD (*n* = 3 independent experiments). Data are presented as the mean ± SD. Statistical analysis was determined by 2-way ANOVA followed by a multiple-comparison test. (**I**) Immunoblot analysis of key enzymes in proline synthesis in GSCs (GSC23 and GSC387) with or without *SPIN1* KD. (**J**) Immunoblot analysis of SPIN1 in GSCs (GSC23 and GSC387) with or without *FAM3C* KD. (**K**) Immunoblot analysis of nuclear and cytoplasmic fractions of GSCs (GSC23 and GSC387) with or without *FAM3C* KD. (**L**) HEK-293 cells were cotransfected with FLAG-tagged FAM3C and either MYC-tagged full-length SPIN1 or a MYC-tagged C-terminal deletion mutant of SPIN1 (MYC-SPIN1-ΔC23-term). Cell lysates were subjected to IP using an anti-FLAG antibody. The resulting precipitates and corresponding whole-cell lysates (input) were analyzed by Western blotting using antibodies against the MYC-tag, FLAG-tag, and FAM3C.

The function of FAM3C has been predominantly considered to be mediated by its secretory/paracrine effects. However, the cell-specific expression and effects on proline metabolism suggested that it may have a cell-intrinsic function potentially mediated through intracellular functions. Following the hypothesis that FAM3C acts in GSCs as a cytoplasmic protein, we overexpressed FAM3C and conducted a pull-down assay using a FAM3C antibody, followed by MS analysis (Supplemental Figure 2A). MS identified potential interactions between FAM3C and multiple proteins (Figure 4A). Several potentially important target proteins were among those with the highest association with FAM3C; we prioritized spindlin 1 (SPIN1), given its role in transcriptional regulation and chromatin remodeling (35). SPIN1 is a chromatin-associated protein that specifically recognizes and binds to histone 3 lysine 4 trimethyl lysine 9 trimethyl (H3K4me3K9me3) (36–38). As a multivalent histone modification reader, SPIN1 is implicated in diverse cellular processes, including the regulation of ribosomal RNA transcription, chromosome segregation, and tumorigenesis (39). The interaction between FAM3C and SPIN1 was validated in 2 GSC lines using IP followed by western blot analysis (Figure 4B). In GSCs, H3K4me3 peaks were predominantly located near promoters, especially at transcription start sites (Figure 4C and Supplemental Figure 2, B and C) (40). Genes marked by H3K4me3 peaks were intersected with genes downregulated following FAM3C KD, and then overlapping genes were subjected to KEGG pathway enrichment analysis (Figure 4D). Genes marked by H3K4me3 and regulated by FAM3C were enriched for arginine and proline metabolism (Figure 4E). H3K4me3 ChIP-seq of GSCs and DGCs showed preferential modifications of the proline synthesis enzymes, P5CS, PYCR1, and PYCR2 in GSCs (Figure 4F). Concordant with the regulation of proline levels in GSCs, SPIN1 KD attenuated the expression of proline biosynthesis genes at both transcript and protein levels (Figure 4, G–I). ChIP-seq in GSC23 identified prominent SPIN1 occupancy peaks in the promoter regions of key proline synthesis enzymes, including ALDH18A1 (P5CS), PYCR1, and PYCR2 (Supplemental Figure 2D). To quantitatively validate these findings, we performed ChIP-qPCR, which revealed enrichment of SPIN1 at the promoters of these proline biosynthetic genes compared with the IgG control in both GSC23 (Supplemental Figure 2E) and GSC387 (Supplemental Figure 2F).

To interrogate FAM3C regulation of SPIN1, we transduced GSCs with shFAM3Cs or shCONT. Targeting FAM3C did not alter *SPIN1* mRNA levels measured by quantitative real-time (qRT-PCR) (Supplemental Figure 2, G and H) but reduced SPIN1 protein levels measured by immunoblotting (Figure 4J), suggesting that FAM3C modulated SPIN1 expression posttranscriptionally. Following FAM3C silencing, SPIN1 levels were reduced in both the nucleus and cytoplasm, as shown by nuclear and cytoplasmic fractionation (Figure 4K). The known nuclear localization signal (NLS) for SPIN1 is located within the C-terminal 23 amino acids (41). Deletion of the NLS did not impair the interaction between FAM3C and SPIN1 (Figure 4L).

**Figure 5:**
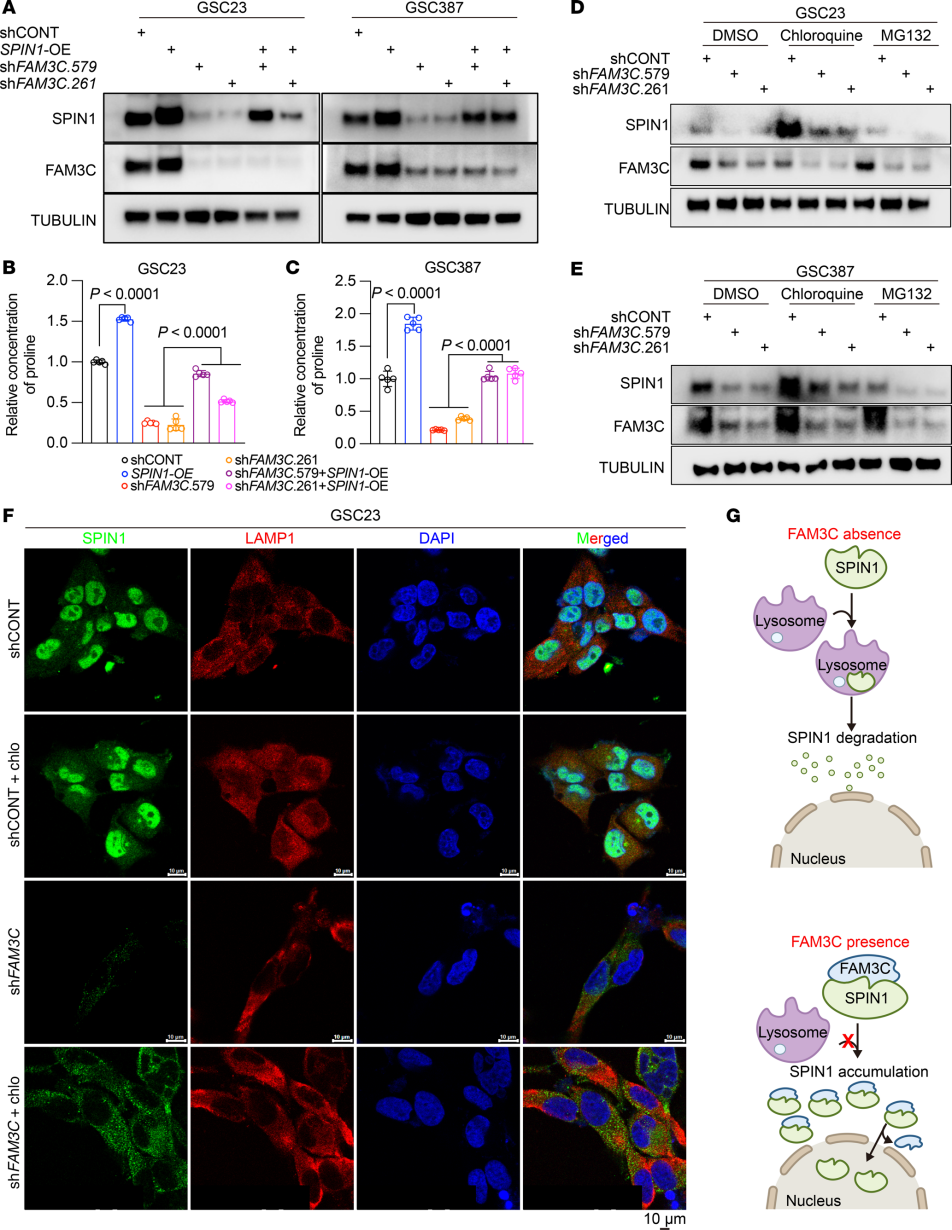
FAM3C regulates SPIN1 stability and nuclear translocation to fuel proline biosynthesis. (**A**) Immunoblot analysis of SPIN1 and FAM3C expression in GSCs (GSC23 and GSC387) with or without *FAM3C* KD, with or without *SPIN1* OE, or in combination with *FAM3C* KD and *SPIN1* OE. (**B** and **C**) Relative intracellular proline levels (*n* = 5 biologically independent samples) in GSC23 (**B**) and GSC387 (**C**) GSCs under the different treatment conditions in **A**. Data are presented as the mean ± SD. Statistical analysis between shCONT and *SPIN1*-OE was performed using a 2-tailed, unpaired *t* test. Statistical analysis between sh*FAM3C* and sh*FAM3C* plus *SPIN1*-OE was performed using ordinary 1-way ANOVA. (**D** and **E**) Immunoblot analysis of SPIN1 and FAM3C expression of GSC23 (**D**) and GSC387 (**E**) GSCs with or without *FAM3C* KD, after treatment with chloroquine to inhibit lysosomal degradation. (**F**) Representative images of SPIN1 (green) and a lysosomal marker, LAMP1 (red), in GSC23 GSCs. DAPI (blue). Scale bars: 10 μm. (**G**) Schematic diagram of the interaction between FAM3C and SPIN1 proteins to prevent the lysosomal degradation of SPIN1 and promote the nuclear localization of SPIN1.

To determine whether FAM3C influences proline biosynthesis in GSCs through regulation of SPIN1, we overexpressed SPIN1 alone and in combination with FAM3C silencing, which confirmed that targeting of FAM3C reduced SPIN1 protein levels (Figure 5A). FAM3C silencing reduced proline levels to less than half baseline levels, whereas SPIN1 overexpression (OE) increased proline levels (Figure 5, B and C). Reintroduction of SPIN1 in FAM3C-silenced cells restored proline levels to near those of the control group (Figure 5, B and C), suggesting that SPIN1 is downstream from FAM3C in regulating proline synthesis in GSCs. MS31 is an inhibitor that disrupts the interaction between SPIN1 and H3K4me3 (42). We determined its IC_50_ at both 24 and 48 hours (Supplemental Figure 2I). We found that MS31 suppressed proline synthesis in GSCs at both time points (Supplemental Figure 2, J and K). These findings provide evidence that SPIN1 regulated proline synthesis within these cells. As FAM3C regulates SPIN1 at a posttranscriptional level, we interrogated potential regulatory mechanisms, starting with posttranslational protein stability. We transduced GSCs with either shCONT or shFAM3C, along with vehicle control, the lysosomal inhibitor chloroquine, or the proteosomal inhibitor MG132, and then measured SPIN1 protein levels to determine whether SPIN1 levels could be rescued. Chloroquine increased SPIN1 levels under control conditions and upon targeting of FAM3C, in contrast to MG132, suggesting that FAM3C may stabilize SPIN1 by reducing its lysosomal degradation (Figure 5, D and E). Using immunofluorescence, FAM3C KD inhibited SPIN1 levels, accompanied by colocalization with LAMP1, a lysosomal marker; furthermore, while chloroquine treatment alone increased nuclear SPIN1 expression, this effect was lost upon FAM3C KD, which instead caused SPIN1 to accumulate in the cytoplasm, but it did not cause the nuclear localization of SPIN1 seen in shCONT cells (Figure 5F and Supplemental Figure 3). These data imply that FAM3C regulates both SPIN1 stability (via a lysosome-dependent mechanism) and nuclear translocation and that FAM3C is required to prevent lysosomal sequestration of SPIN1 and enable its nuclear translocation (Figure 5G).

### FAM3C depletion induces loss of redox homeostasis.

Oxidative stress arises when the generation of ROS exceeds the cellular ability to neutralize them with antioxidants (43). Excessive ROS levels cause damage to DNA, proteins, and lipids, eventually leading to cell death (44, 45). Metabolic adaptations contribute to the modulation of redox homeostasis (46). Proline metabolism contributes to the regulation of intracellular redox homeostasis (47). During proline biosynthesis, net NAD(P)^+^ is generated, whereas proline can react directly with hydroxyl radicals (·OH) to form hydroxyproline, acting as a direct chemical scavenger of ROS (Supplemental Figure 4A) (48, 49). This interconversion, known as proline cycling, facilitates redox equilibrium and serves as a protective mechanism against oxidative stress mediated by ROS (Supplemental Figure 4A) (50).

**Figure 6:**
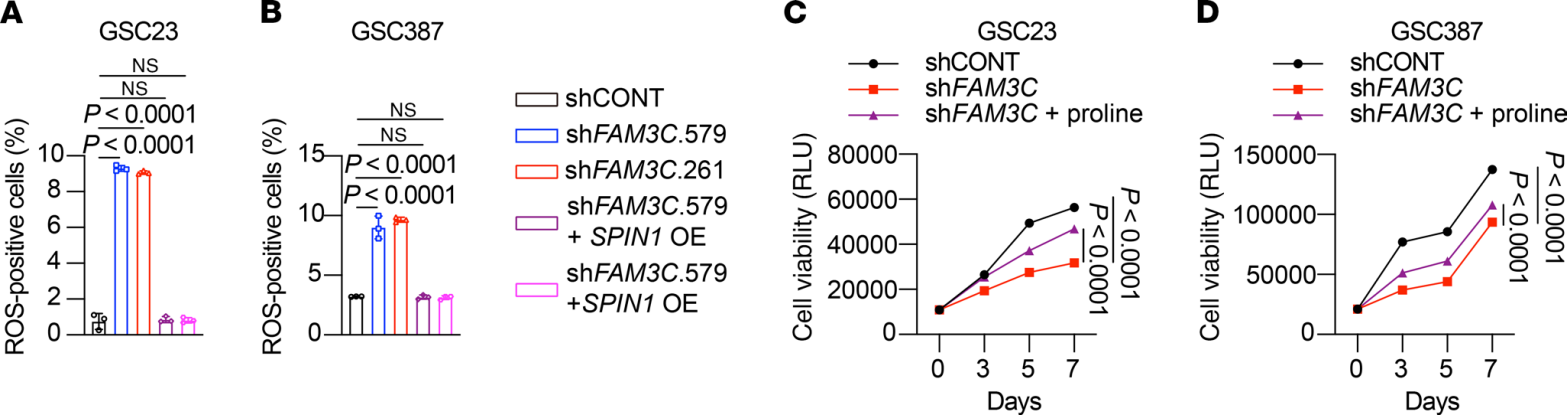
FAM3C maintains redox homeostasis. (**A** and **B**) Quantification (*n* = 3 biologically independent samples per group) of ROS flow cytometric analysis of GSC23 (**A**) and GSC387 (**B**) GSCs. The cutoff used to define ROS^+^ and ROS ^–^ signal is 10^3^ on the logarithmic scale. (**C** and **D**) Cell viability (*n* = 3 independent experiments) of GSC23 (**C**) and GSC387 (**D**) GSCs with or without *FAM3C* KD or in combination with *FAM3C* KD and proline supplementation. Data are presented as the mean ± SD in **A**–**D**. Significance was determined by 1-way ANOVA followed by a multiple-comparison test (**A** and **B**) and 2-way ANOVA followed by a multiple-comparison test (**C** and **D**).

As ROS are associated with cancer stem cell biology, we hypothesized that FAM3C and proline function, at least partially, through ROS regulation. To investigate the relationship between proline metabolism and ROS, we utilized ROS transcriptomic signatures defined by the ratio of superoxide (O_2_^–^) to hydrogen peroxide (H_2_O_2_) (51, 52). In GBM, proline metabolism was enriched in samples with low ROS transcriptomic signatures, in both TCGA and CGGA datasets (Supplemental Figure 4, B and C). Analysis of a single-cell database revealed that GSCs had lower ROS index values compared with other GBM cells (Supplemental Figure 4D) (53). In 3 paired GSC and DGC samples, GSCs consistently showed lower ROS indices (Supplemental Figure 4E). Targeting FAM3C expression induced ROS levels in two GSCs, which was rescued by SPIN1 OE. These findings suggest that FAM3C and SPIN1 modulate oxidative stress in GSCs (Figure 6, A and B, and Supplemental Figure 4, F and G). After knocking down FAM3C, supplementing the GSCs with 150 μM proline for 24 hours partially rescued ROS levels (Supplemental Figure 4, H–K) and cell viability (Figure 6, C and D), suggesting that FAM3C regulated ROS levels by influencing proline synthesis.

To determine whether hydroxyproline recapitulates the ROS-suppressive effects of proline, we transduced GSCs with either shCONT or shFAM3C and then treated them with PBS or either proline or hydroxyproline (200 μM) for 48 hours. In control we observed that GSCs, neither proline nor hydroxyproline supplementation altered the baseline ROS levels, which remained low (Supplemental Figure 5, A–C). In FAM3C-deficient GSCs, proline supplementation reduced ROS levels, whereas hydroxyproline supplementation failed to demonstrate ROS-suppressive effects (Supplemental Figure 5, A–C). These results suggest that the ROS-scavenging effect was not a general property of proline-related metabolites but was specifically linked to proline oxidation itself. In GSC23 treated with the mitochondrial ROS inducer MitoPQ, the fractional contribution of ^13^C-proline to 4-hydroxyproline increased (Supplemental Figure 5D). As the concentration of MitoPQ increased (0 μM to 50 μM), the NADPH/NADP^+^ ratio decreased (Supplemental Figure 5, E and F). This suggests that under oxidative stress, the cellular pool of proline is consumed by its direct reaction with ROS.

### FAM3C promotes GSC proliferation and predicts poor prognosis.

**Figure 7:**
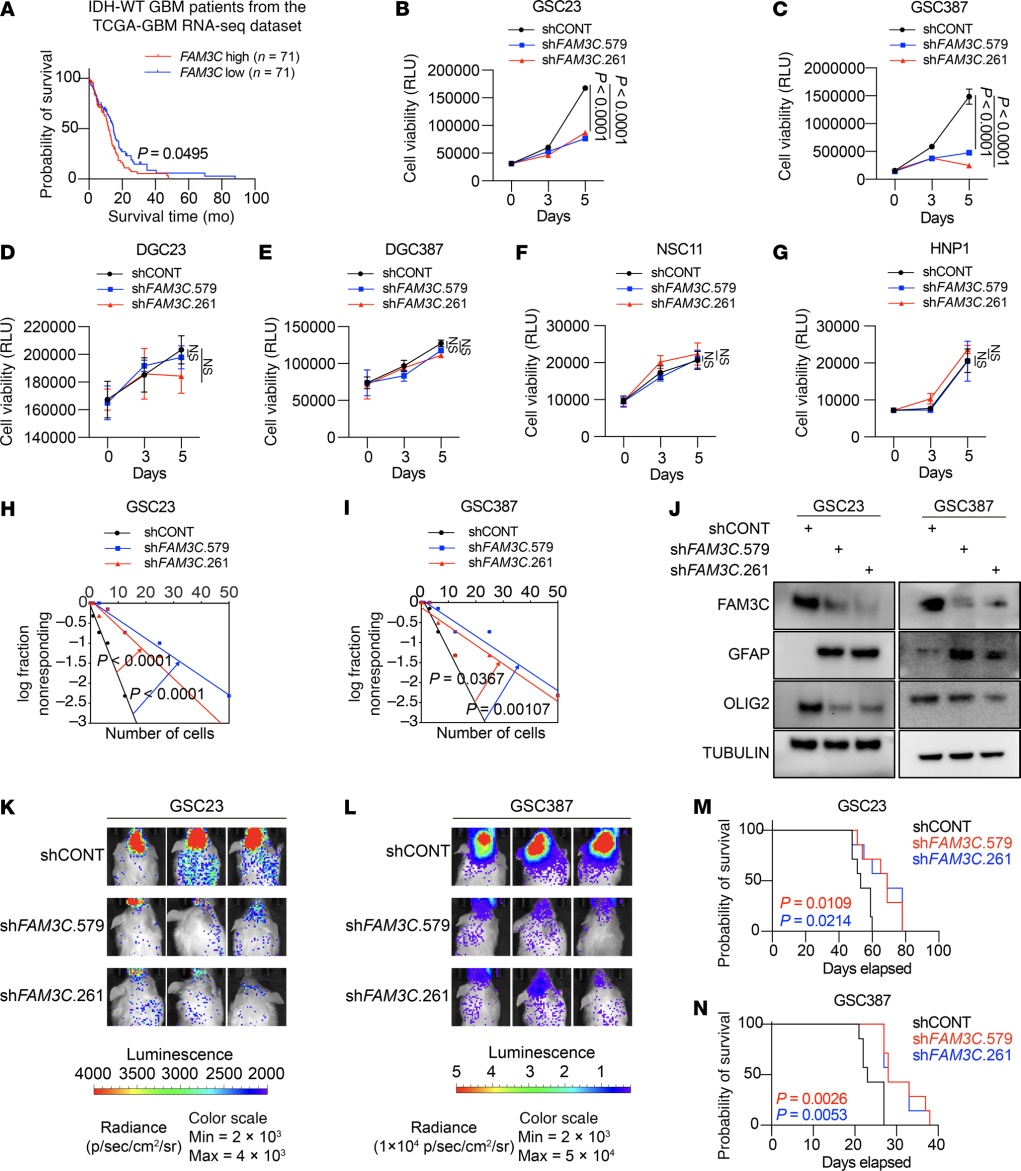
FAM3C promotes GSC proliferation and portends a poor prognosis. (**A**) Kaplan-Meier survival curves for TCGA_GBM based on *FAM3C* mRNA expression. The top 50% and the bottom 50% are defined as high (*n* = 71) and low (*n* = 71) groups, respectively. Median survival time (MST) for the FAM3C^hi^ group = 11.8 months; MST for FAM3C^lo^ group = 14.7 months. (**B**–**G**) Cell viability (*n* = 3 independent experiments) in GSCs (*n* = 3 independent experiments) (**B** and **C**), DGCs (*n* = 5 independent experiments) (**D** and **E**), and NSCs (*n* = 5 independent experiments) (**F** and **G**) with or without *FAM3C* KD. (**H** and **I**) Extreme limiting dilution assay (*n* = 10 independent experiments) in GSC23 (**H**) and GSC387 (**I**) GSCs with or without *FAM3C* KD. (**J**) Immunoblot analysis of FAM3C, GFAP, and OLIG2 expression in GSC23 and GSC387 GSCs with or without *FAM3C* KD. (**K** and **L**) Representative in vivo bioluminescence images of tumor-bearing mice derived from GSC23 (**K**) and GSC387 (**L**) GSCs with or without *FAM3C* KD on day 21. Representative images from 7 mice are shown. (**M** and **N**) Kaplan-Meier survival curves of mice bearing the indicated xenografts. *n* = 7 mice per group for GSC23 cells (**M**) and GSC387 cells (**N**). In **B**–**G**, data are presented as the mean ± SD. Statistical significance was determined by log-rank test (**A**, **M**, and **N**), 2-way ANOVA followed by a multiple-comparison test (**B**–**G**), and the 2-tailed likelihood ratio test (**H** and **I)**.

FAM3C has been associated with a poor prognosis in all gliomas (28), but this report did not account for WHO tumor classification. In TCGA cohort, higher FAM3C expression was associated with a poorer prognosis for patients with IDH WT GBM (Figure 7A). Targeting FAM3C reduced GSC viability (Figure 7, B and C), with a minimal effect on DGC viability (Figure 7, D and E) or NSCs (Figure 7, F and G), supporting a specific dependency on FAM3C in GSCs. Targeting FAM3C expression inhibited GSC self-renewal, as measured by decreased sphere formation in extreme limiting dilution assays (Figure 7, H and I). Depletion of FAM3C reduced the GSC sphere size (Supplemental Figure 6, A–C) and 5-ethynyl-2′-deoxyuridine (EdU) incorporation (Supplemental Figure 6, D–G). Targeting FAM3C reduced GSC invasion (Supplemental Figure 6, H–J).

FAM3C KD in GSCs decreased the levels of OLIG2 — a key marker of stemness — and increased the expression of GFAP, a marker of DGCs (Figure 7J). To determine whether the loss of stemness in FAM3C-deficient cells is causally linked to elevated ROS levels, we performed rescue experiments using the ROS scavenger *N*-acetylcysteine (NAC) in both GSC23 and GSC387. Flow cytometric analysis confirmed that FAM3C KD increased ROS levels in both GSC lines, which was effectively suppressed by NAC treatment in a concentration-dependent manner (0.02–20 μM) (Supplemental Figure 7, A and B). We then assessed the effect of this ROS scavenging on GSC maintenance. In GSCs with FAM3C KD, treatment using low-to-moderate concentrations of NAC (0.02–20 μM) showed that increasing concentrations of NAC scavenged excess ROS, leading to progressive recovery of stemness markers, including OLIG2 and CD133, and a decrease in the differentiation marker GFAP (Supplemental Figure 7, C and D). In contrast, NAC treatment at these concentrations of control GSCs (i.e., transduced with shCONT) did not affect stemness (Supplemental Figure 7, C and D). These results support the concept that the loss of stemness following FAM3C KD was driven, at least in part, by elevated ROS levels. We also observed that very high concentrations of NAC (200 μM) led to a decline in stemness markers and an increase in GFAP expression, regardless of GSC group (Supplemental Figure 7, C and D). This biphasic response was likely due to ROS-independent effects of NAC at higher concentrations. NAC serves as a precursor that boosts glutathione (GSH) biosynthesis (54). Excessive GSH can conjugate with fumarate, leading to reduced fumarate levels and decreased succination of PTEN at the C211 residue (54). This allows PTEN to bind to MMS19, which interferes with iron-sulfur cluster assembly, ultimately impairing GSC self-renewal and proliferation (54).

The gold standard of GSCs is in vivo tumor growth. To evaluate the role of FAM3C in GSC tumor initiation in vivo, 2 patient-derived GSC lines were transduced with either a nontargeting shCONT or 1 of 2 distinct shRNAs specifically silencing FAM3C. These cells were then intracranially implanted into immunodeficient mice. Silencing FAM3C resulted in slower tumor growth (Figure 7, K and L, and Supplemental Figure 7, E and F) and reduced tumor burden (Supplemental Figure 7, G and H) and extended survival compared with the shCONT group (Figure 7, M and N).

### Tucatinib disrupts the interaction between FAM3C and SPIN1.

**Figure 8:**
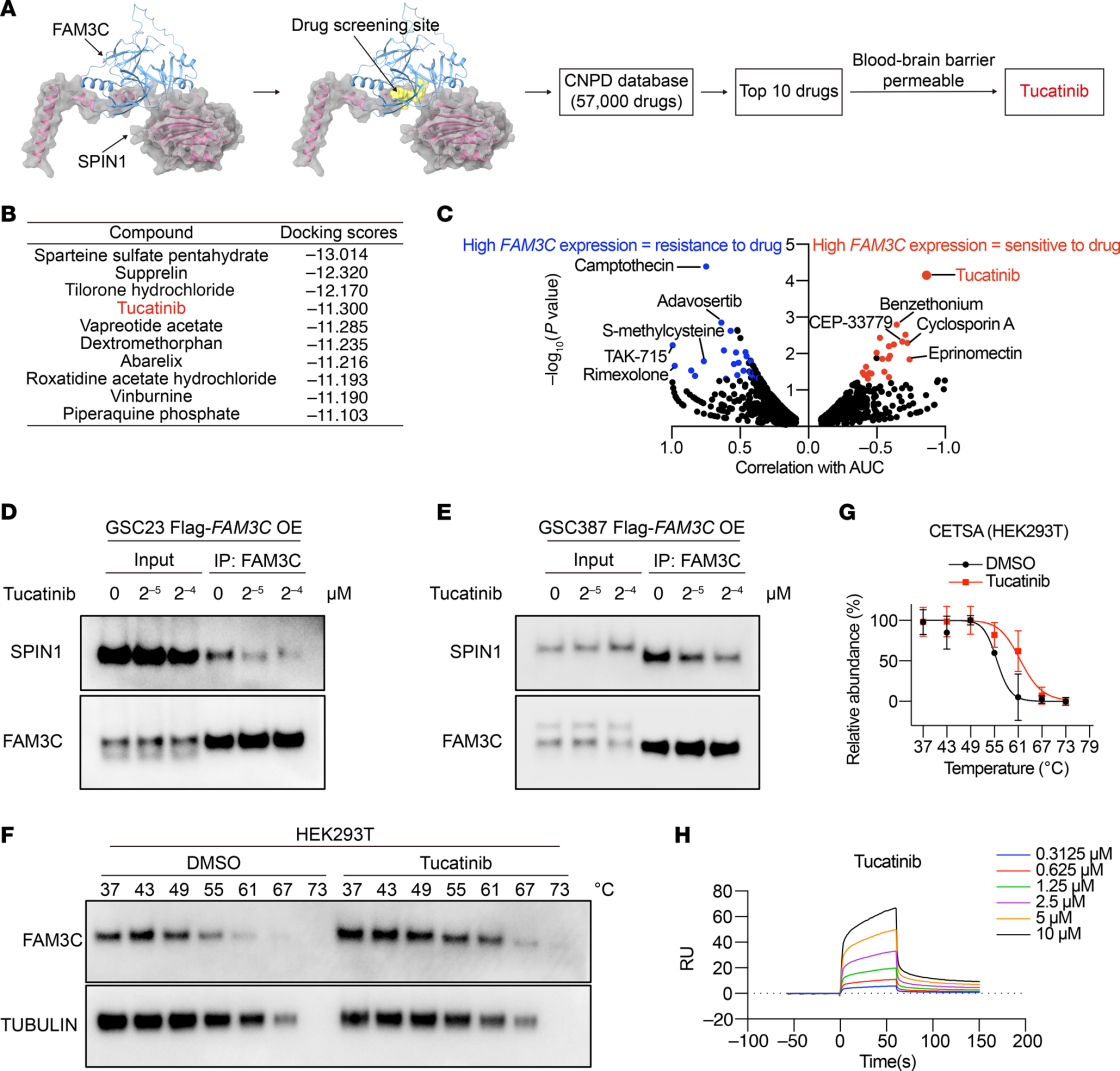
Tucatinib disrupts the interaction between FAM3C and SPIN1. (**A**) Drug screening strategy based on the binding interface between FAM3C and SPIN1. (**B**) The 10 compounds with the highest docking scores identified on the basis of the drug screening strategy in **A**. Docking scores below –6 were considered indicative of effective binding. (**C**) Therapeutic efficacy prediction of drugs for FAM3C (Methods). The blue dot shows the top resistance drug, and red dots show the top sensitive drugs for high FAM3C expression. Two-tailed Pearson correlation was used for statistical analysis. (**D** and **E**) Immunoblot analysis of FAM3C IP was performed on GSC23 (**D**) and GSC387 (**E**) cells overexpressing Flag-FAM3C after treatment with gradually increasing concentrations of tucatinib (0 μM, 2^–5^ μM, 2^–4^ μM) for 6 hours. (**F** and **G**) Immunoblot analysis (**F**) and thermal shift curves (**G**) of FAM3C from a cellular thermal shift assay (CETSA) in HEK293T cells pretreated with 2^–5^ μM tucatinib (*n* = 3 independent experiments). Data are presented as the mean ± SD. CETSA, cellular thermal shift assay. (**H**) SPR was used to analyze binding affinities and kinetic parameters between FAM3C and tucatinib**.**

To facilitate the potential clinical translation of our findings, we explored repurposing of existing compounds in the Chinese Natural Product Database (CNPD) to inhibit FAM3C activity, thereby disrupting SPIN1 stabilization and maintenance of GSCs. First, we performed molecular docking predictions using the crystal structures of FAM3C and SPIN1 obtained from the Protein Data Bank (PDB) (Figure 8A). Based on predicted BSs between FAM3C and SPIN1, approximately 57,000 compounds from the CNPD underwent in silico screening with a cutoff score of –6 for effective binding (Figure 8A). The top 10 compounds with the highest docking scores were identified (Figure 8B), among which tucatinib is FDA approved and also the only known blood-brain barrier–permeable (BBB-permeable) compound (55). A dominant concern in neuro-oncologic drug development is delivery across the neurovascular unit (i.e., blood-tumor barrier). Sparteine sulfate pentahydrate and tilorone hydrochloride are macrocyclic complex structures; supprelin is a bridged ring compound (Supplemental Figure 8, A–C). In contrast, tucatinib has a simpler structure and superior docking activity (Supplemental Figure 8D). To prioritize the choice of compound for investigation, we analyzed pharmacogenomic profiles from the Cancer Therapeutics Response Portal (CTRP) to uncover drug sensitivities linked to FAM3C expression (56, 57). Compounds exhibiting AUC values correlated with FAM3C expression in brain tumor–derived cell lines were selected and categorized on the basis of their AUC distributions. Tucatinib emerged as the leading candidate agent for *FAM3C*-predicted sensitivity ranked by *P* value (Figure 8C and Supplemental Figure 8, E–J). Thus, we selected tucatinib for further investigation as a therapeutic agent targeting FAM3C-based druggable dependencies.

Increasing concentrations of tucatinib progressively diminished the binding affinity between FAM3C and SPIN1 in 2 GSC lines (Figure 8, D and E). Tucatinib shifted the thermal stability of FAM3C protein, confirming a direct interaction between FAM3C and tucatinib (Figure 8, F and G). Human FAM3C protein was subjected to surface plasmon resonance (SPR) with tucatinib. We found that tucatinib directly bound to human FAM3C protein with a *K_D_* of 5.12 μM (Figure 8H).

**Figure 9:**
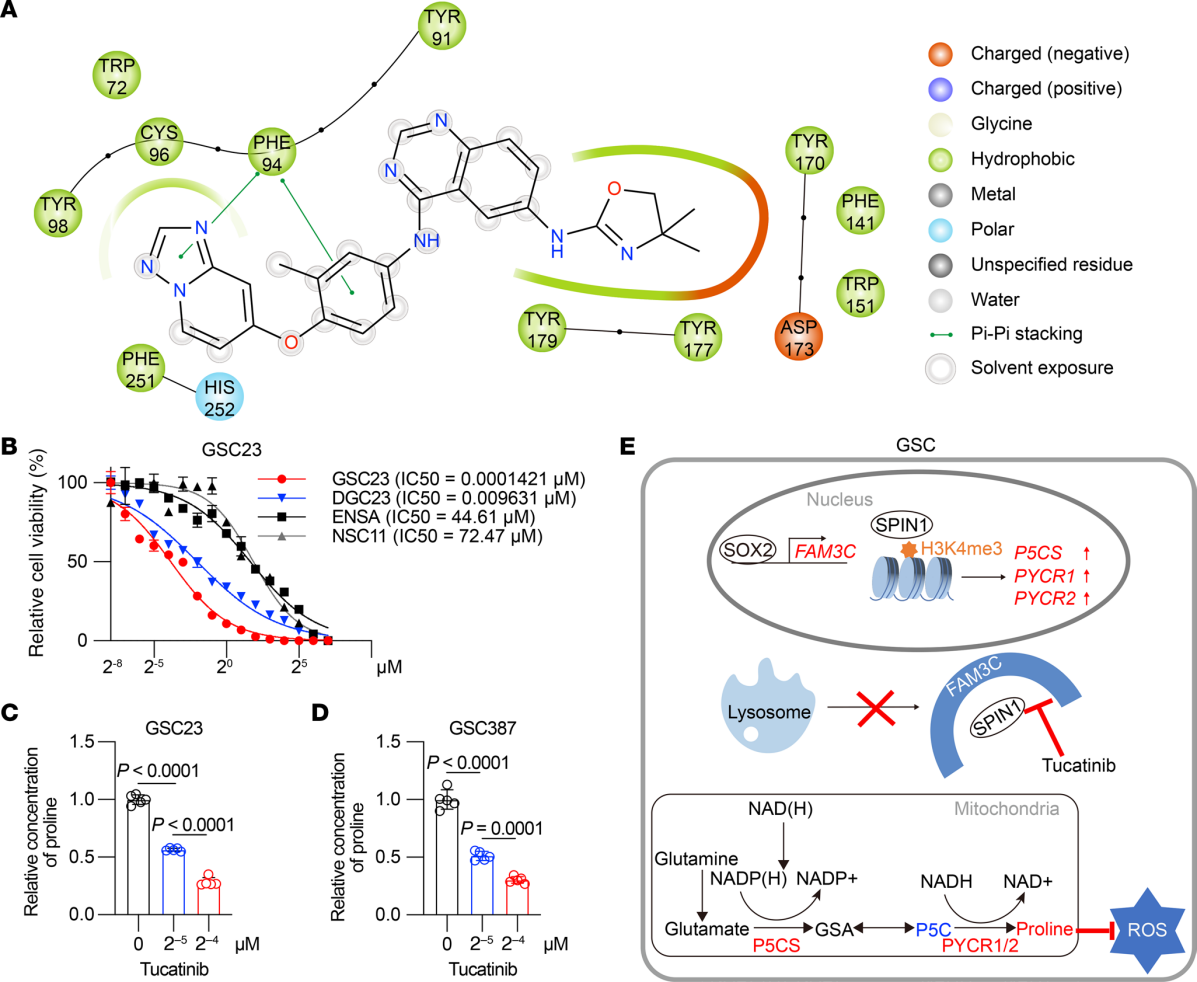
Tucatinib impairs proline biosynthesis and inhibits the proliferation of GSCs. (**A**) 2D ligand interaction diagrams for tucatinib. (**B**) Dose-response curves of tucatinib in GSC23 cells, DGC23 cells, and NSCs (NSC11 and ENSA) (*n* = 3 independent experiments). Data are presented as the mean ± SD. (**C** and **D**) The intracellular proline levels (*n* = 5 biologically independent samples) of GSC23 (**C**) and GSC387 (**D**) GSCs after treatment with gradually increasing concentrations of tucatinib (0 μM, 2^–5^ μM, 2^–4^ μM) for 6 hours. Data are presented as the mean ± SD. Statistical analysis was performed using 1-way ANOVA followed by multiple comparison. (**E**) Schematic of this study. In brief, SOX2 drives FAM3C expression in GSCs, where FAM3C interacts with SPIN1 to upregulate proline biosynthetic enzymes (P5CS and PYCR1/2). Elevated proline synthesis depletes cellular ROS, supporting GSC maintenance. This signaling axis can be pharmacologically disrupted by Tucatinib, which targets the FAM3C-SPIN1 interaction.

As for SPIN1, at a biochemical level, in silico analysis revealed that residue F94 was essential for the binding of SPIN1 with tucatinib (Figure 9A). Tucatinib reduced GSC viability, with a lower IC_50_ compared with both matched DGCs and NSCs (NSC11 and ENSA) (Figure 9B and Supplemental Figure 8K). Tucatinib induced a concentration-dependent decrease in proline concentration in the GSCs (Figure 9, C and D), suggesting that tucatinib inhibits proline synthesis in GSCs by interfering with the biological functions of FAM3C and SPIN1. An increase in tucatinib concentration was found to be directly correlated with an increased proportion of ROS^+^ cells in GSCs (Supplemental Figure 8, L and M).

In conclusion, stem-like tumor cells specifically enriched proline as a result of increased expression of proline biosynthesis enzymes, including P5CS and PYCR1/2, which are associated with epigenetically active H3K27ac and H3K4me3 marks. The core stem cell factor SOX2 preferentially promoted transcriptional upregulation of FAM3C in GSCs. FAM3C enhanced the stability of SPIN1 — a reader of H3K4me3 epigenetic marks — by preventing its degradation via the lysosomal pathway and assisting its nuclear localization, thereby permitting increased transcription of proline synthesis in GSCs and contributing to enhanced ROS clearance. Taken together, these findings highlight the therapeutic potential of targeting the FAM3C/SPIN1 axis in GBM and identify tucatinib as a promising agent with a broad therapeutic window (Figure 9E).

## Discussion

Amino acids hold special places in the pantheon of metabolites in cancer biology. Appropriate levels of amino acids are critical for generating proteins for rapidly dividing tumor cells, but an even more complex landscape is being revealed for amino acids given the unique roles that different amino acids play in fate determination in tumor initiation and progression. Against this backdrop, proline, a nonessential amino acid characterized by its unique cyclic configuration, plays a pivotal role in cellular metabolism. Its importance is increasingly acknowledged across various physiological processes — including energy metabolism, redox balance, and programmed cell death — as well as in pathological conditions (58). Metabolic analysis of sphere-forming canine mammary tumor cells, potentially representing cancer stem cells, shows elevated concentrations of multiple amino acids, including proline (59). Osteosarcoma cells exhibiting stem-like properties (60) also contain higher levels of proline and arginine (61). Supplementing DMEM with proline supports the maintenance of pluripotency in embryonic stem cells (61). Moreover, the expression levels of key enzymes involved in proline biosynthesis, including P5CS, PYCR1, and PYCR2, are upregulated in multiple cancer types (11, 19, 62, 63). In our study, proline levels were elevated in GSCs compared with DGCs (Figure 1) in association with P5CS, PYCR1, and PYCR2 (Figure 1). These findings further support the critical role of proline metabolism in maintaining the stemness of cancer stem cells.

However, the regulatory mechanisms underlying the upregulation of these key enzymes in GSCs remain largely unexplored. Here, we uncovered a regulatory pathway governing proline synthesis. We discovered that FAM3C plays a critical role in proline biosynthesis in GSCs. To date, FAM3C has been largely considered in the context of its secreted function, as it has also been designated as ILEI and activates multiple intracellular oncogenic pathways. Here, we found a mechanism for FAM3C function based on an intracellular, cell-autonomous reprogramming of tumor cell metabolism. GSCs express high levels of FAM3C, both secreted and intracellular, but the functional regulation of proline synthesis occurs through cytoplasmic stabilization of SPIN1, which in turn promotes the elevated transcription of ALDH18A1, PYCR1, and PYCR2 (Figure 9E). FAM3C contributes to tumor progression in other cancers (25, 27, 31). In gliomas, FAM3C is highly expressed and contributes to tumor cell proliferation, potentially through regulation of Notch signaling (28, 64). FAM3C promotes metabolic reprogramming through epigenetic effects occurring via an intracellular function.

SPIN1 is an evolutionarily conserved protein that recognizes histone methylation marks (65). Structurally, SPIN1 contains 3 tandem Tudor-like domains, with the second domain specifically binding to H3K4me3 (36), a histone modification typically associated with promoters of actively transcribed or transcriptionally poised genes (37, 66–68). Through this recognition, SPIN1 regulates downstream gene expression (38). Elevated levels of SPIN1 have been observed across multiple cancer types (69–72). Silencing SPIN1 expression or targeting it with small-molecule inhibitors inhibits cancer cell proliferation in vitro and suppresses tumor growth in xenograft models (38). In our study, we found that SPIN1 directly regulated the transcription of genes involved in proline biosynthesis, thereby influencing intracellular proline levels (Figure 4, G–I). Molecular docking (Figure 9A) identified F94 as a critical residue for FAM3C-SPIN1 interaction, a site distant from the C-terminal NLS (the last 23 amino acids). Although our co-IP experiments using an NLS-truncated SPIN1 mutant demonstrated that the NLS was not required for FAM3C binding, we also found that, while chloroquine treatment alone increased nuclear SPIN1 expression, this effect was lost upon FAM3C KD, which instead caused SPIN1 to accumulate in the cytoplasm, but did not cause the nuclear localization of SPIN1 seen in shCONT cells (Figure 5F and Supplemental Figure 3). These data imply that FAM3C regulates both SPIN1 stability (via a lysosome-dependent mechanism) and nuclear translocation and that FAM3C is required to prevent lysosomal sequestration of SPIN1 and enable its nuclear translocation.

ROS have a dual role in tumor progression. On the one hand, ROS-driven pathways fuel tumor cell proliferation and therapeutic resistance in various cancers (52). For instance, hydrogen peroxide (H_2_O_2_) oxidatively modifies prolyl hydroxylase domain protein 2 (PHD2), leading to the stabilization of HIF-1, a major regulator of angiogenesis and metastatic progression (73). Moreover, ROS can trigger the activation of NF-κB through protein kinase D1 and enhance proliferative signals via the EGF receptor (EGFR) activation (74). On the other hand, excessive ROS contribute to genomic instability by inducing various forms of DNA damage, such as strand breaks, point mutations, and base mismatches (75). In addition to compromising nuclear DNA integrity, ROS can also target mitochondrial DNA, disrupting electron transport chain complex I function (76). These oxidative lesions frequently involve modifications to nucleotide bases and sugar backbones, ultimately interfering with DNA repair fidelity and genomic maintenance (77). In GSCs, low levels of ROS are crucial for maintaining stemness and their ability to resist therapy (78), which is consistent with our findings (Supplemental Figure 4, D and E). Our study further revealed a direct link between proline metabolism and ROS regulation (Supplemental Figure 4, B and C). FAM3C and SPIN1 collaboratively modulated proline biosynthesis, thereby indirectly enhancing the capacity of GSCs to mitigate ROS accumulation (Figure 6, A and B, and Supplemental Figure 4, F and G). This provides an alternative mechanistic explanation for how GSCs maintain low intracellular ROS levels.

Using multiple screening approaches, we identified tucatinib as a potential inhibitor that specifically disrupts the interaction between FAM3C and SPIN1, highlighting its promise as a therapeutic candidate for GBM treatment (Figure 8). Tucatinib is an orally administered tyrosine kinase inhibitor approved by the FDA for the treatment of breast and colorectal cancers and is characterized by its strong selectivity for the HER2 kinase domain and minimal interaction with EGFR. Tucatinib demonstrates limited toxicity, efficiently penetrates the BBB, and shows antitumor efficacy in patients with HER2^+^ metastatic breast cancer who have undergone extensive prior treatment (79–81). Tucatinib exerted selective cytotoxic effects on GSCs, while displaying weaker activity against DGCs and NSCs (Figure 9B and Supplemental Figure 8K). Expression of *ERBB2*, the gene encoding HER2, did not differ between GSCs and DGCs (data not shown), suggesting that the therapeutic efficacy of tucatinib in GSCs may involve targets other than HER2. Future in vivo animal studies and clinical trials will be necessary to determine the optimal therapeutic dosage and to validate the efficacy and safety of tucatinib in the treatment of GBM. In addition, our results suggest that there may be combination strategies to build on tucatinib, including those involving dietary therapies (10). While the SPIN1 inhibitor MS31 is not likely to be translatable to clinical practice, combined targeting with tucatinib and a MS31-like agent may confer a combinatorial benefit.

Collectively, our findings highlight the contribution of proline biosynthesis to the regulation of ROS levels in GSCs. Modulating pivotal enzymes within the proline metabolic network may offer a promising therapeutic avenue for the treatment of GBM.

## Methods

Detailed methods can be found in Supplemental Methods.

### Sex as a biological variable.

Our study used both female and male immunodeficient NOD.Cg-*Prkdc^scid^* *Il2rg^tm1Wjl^*/SzJ (NSG) mice (strain 005557, The Jackson Laboratory), and similar findings are reported for both sexes.

### Animal studies.

NSG mice were used to assess GSC growth in vivo. Briefly, both male and female mice, aged 4–6 weeks, were randomly assigned to each group and maintained in a specific pathogen–free (SPF) animal facility on a 12-hour light/12-hour dark cycle at the University of Pittsburgh. More details on the intracranial tumor injection methods can be found in Supplemental Methods.

### GSC culture.

Patient-derived GSCs were derived from fresh GBM and cultured in Neurobasal media (Gibco, Thermo Fisher Scientific, 21103049) supplemented with B27 without vitamin A (Gibco, 12587010), GlutaMAX (Gibco, 35050061), sodium pyruvate (Gibco, 11360070), penicillin-streptomycin (Gibco, 15140122), recombinant human EGF (rhEGF) (236-EG-01M, R&D Systems), and basic FGF (bFGF) (R&D Systems, 4114-TC-01M). Additional details about patient information can be found in Supplemental Methods.

### Lentivirus production.

For lentivirus production, the shRNA plasmids used in this work were purchased from MilliporeSigma. The shRNA information is listed in Supplemental Table 2. Transfer plasmids were cotransfected with psPax2 (Addgene, 12260) and pMD2G (Addgene, 12259) using PEI (Polysciences, 24765-100) in HEK293T cells. Lentiviruses were collected on days 1, 2, and 3 after transfection.

### qRT-PCR.

RNA was extracted using the Direct-zol RNA Microprep Kit (Zymo Research, R2052) according to the manufacturer’s protocol. cDNA was synthesized with 1 μg RNA by reverse transcription using the High-Capacity cDNA Reverse Transcription Kit (Thermo Fisher Scientific, 4368814). Relative cDNA was quantified by performing qRT-PCR using the Bio-Rad CFX96 Touch Real-Time PCR Detection System with SYBR Green Master Mix (Thermo Fisher Scientific, 4309155).

### Statistics.

Sample sizes were not statistically predetermined but mirrored those typically used in prior investigations (12, 13). For in vivo studies, mice were randomly allocated to their respective experimental cohorts. Similarly, cells designated for nonmurine experiments underwent a comparable randomization process. Data acquisition and subsequent analysis were performed without blinding to the experimental conditions, and all collected data points were included in the analyses. Statistical evaluations were carried out using R, Python, or GraphPad Prism 9 software, with specific details provided in the figure legends For comparisons between two groups, unpaired 2-tailed Student’s *t* tests were used. For multiple group comparisons, 1-way or 2-way ANOVA followed by corrections for multiple comparison were applied. Patient survival or animal survival was analyzed using Kaplan-Meier survival curves, and statistical significance between groups was determined by the log-rank test. Specific statistical tests and sample sizes (*n*) for each experiment are indicated in the respective figure legends. While a normal distribution of data was presumed, this assumption was not formally verified. Unless explicitly stated otherwise, all graphical data represent the mean ± SD. A *P* value below 0.05 was consistently used as the threshold for statistical significance, except where noted otherwise.

### Study approval.

This study complied with all relevant ethics standards and was approved by the ethics committee and IRB at the University of Pittsburgh and the University of North Carolina, Chapel Hill. All murine experiments adhered to the guidelines set by the IACUC of the University of Pittsburgh (protocol 21049014).

### Data availability.

The data supporting the findings of this study are available within the article and in Supplemental Tables 1–7 and Figures 1–9. RNA-seq and ChIP-seq data supporting the findings of this study have been deposited in the Gene Expression Omnibus (GEO) database under (GEO GSE307672). The MS proteomics data have been deposited with the ProteomeXchange Consortium via the PRIDE (82) partner repository (dataset identifier: PXD068325). Human glioma transcriptomics data were derived from TCGA Research Network (http://cancergenome.nih.gov/) and the Chinese Glioma Genome Atlas (CGGA) database (http://www.cgga.org.cn/). Values for all data points in graphs are reported in the Supporting Data Values file. All other data supporting the findings of this study are available from the corresponding author on reasonable request.

## Author contributions

WW and JNR conceived the project, designed the overall experiments, analyzed data, and wrote the manuscript. PZ and DW performed ChIP-qPCR and sample preparation for LC-MS. PZ performed the isotope tracing experiment, cytoplasm and nucleus fractionation, immunofluorescence, and ROS flow cytometric analysis. PZ and DW performed ChIP-seq analysis and protein LC-MS analysis. XW performed Ponceau S staining, Transwell assays, co-IP experiments, drug therapeutic efficacy prediction of FAM3C, and RNA-seq data analysis. PZ and QW performed animal experiments. DL, TH, RW, HL, QW, HM, ST, FY, TD, ZC, and HY analyzed published public data and edited the manuscript. The order of the co–first authors’ names was determined on the basis of the relative scope of their experimental contributions and data analysis.

## Conflict of interest

The authors have declared that no conflict of interest exists.

## Funding support

This work is the result of NIH funding, in whole or in part, and is subject to the NIH Public Access Policy. Through acceptance of this federal funding, the NIH has been given a right to make the work publicly available in PubMed Central.

- NIH grants CA197718, CA238662, CA268634, NS136424, and NS103434 (to JNR).
- Defense Health Agency grant HT9425-23-1-0689 (to JNR).
- American Cancer Society Lisa Dean Moseley Foundation Cancer Stem Cell Consortium (to JNR).
- Start-up funds from the University of Pittsburgh (to JNR).
- Hillman Fellow for Innovative Cancer Research Program (to HY).

## Supplementary Material

Supplemental data

Unedited blot and gel images

Supplemental tables 1-7

Supporting data values

## Version 1 06/01/2026

Electronic publication
