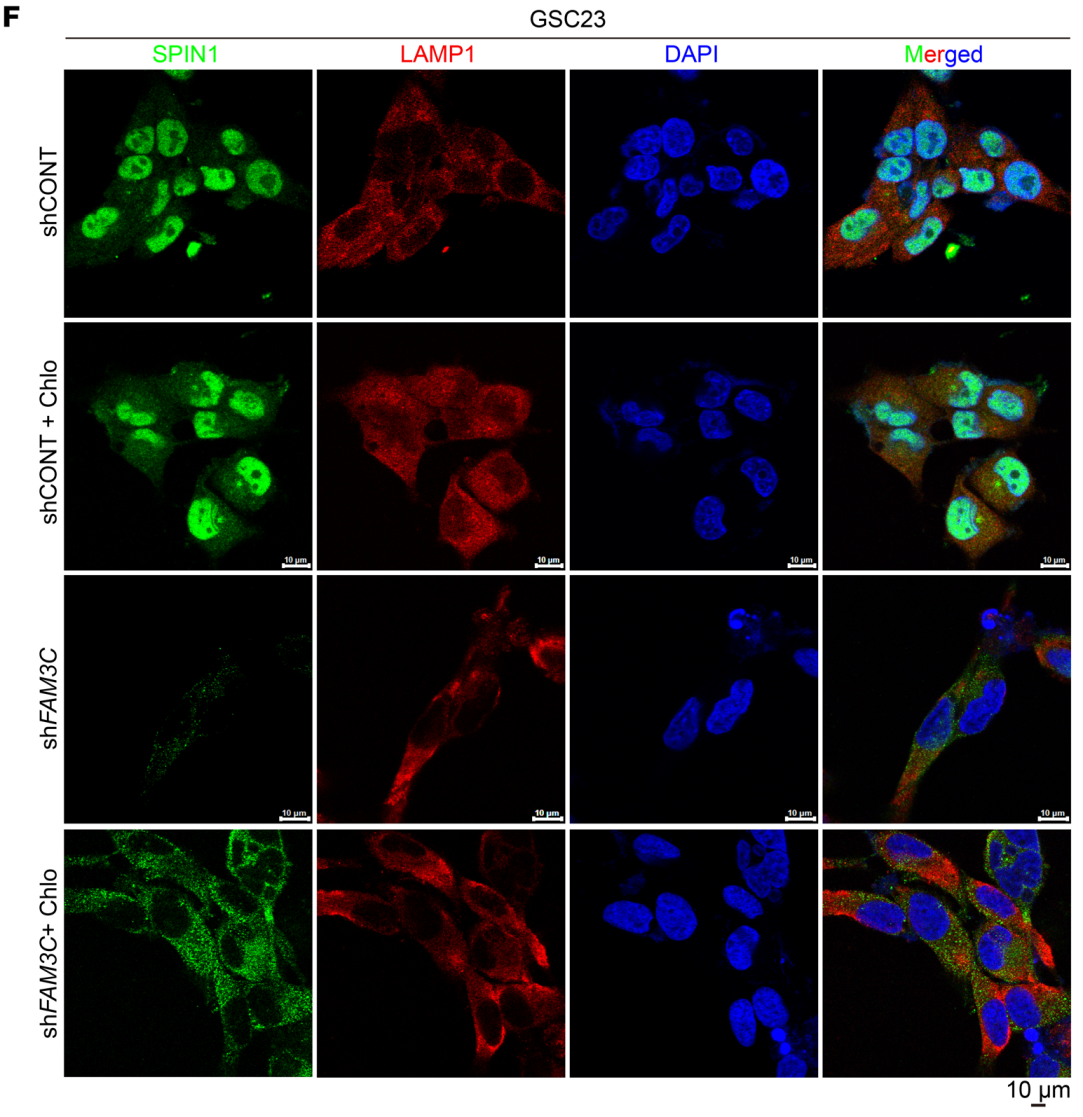# Corrigendum to Cancer stem cells synthesize proline to attenuate oxidative stress

**DOI:** 10.1172/JCI210189

**Published:** 2026-08-03

**Authors:** Weichi Wu, Po Zhang, Donghai Wang, Xujia Wu, Qiulian Wu, Daqi Li, Tengfei Huang, Rui Wang, Huan Li, Hailong Mi, Suchet Taori, Fanen Yuan, Tingting Duan, Zhiye Chen, Huairui Yuan, Jeremy N. Rich

Original citation: *J Clin Invest*. 2026;136(11):e200775. https://doi.org/10.1172/JCI200775

Citation for this corrigendum: *J Clin Invest*. 2026;136(15):e210189. https://doi.org/10.1172/JCI210189

After publication of this article, the authors became aware of a processing error in Figure 5F for the shFAM3c + chlo images. The correct images are shown below. The HTML and PDF versions of the paper have been updated.

The authors regret the error.

## Figures and Tables

**Figure 5F:**