# Supplementary material for: Cancer stem cells synthesize proline to attenuate oxidative stress
Source: J Clin Invest. 2026 Jun 1;136(11):e200775. doi: 10.1172/JCI200775 (PMC13221224; doi:10.1172/JCI200775)
Supplement: Supplemental data [file jci-136-200775-s039.pdf]

Supplemental Material

Cancer stem cells synthesize proline to attenuate oxidative stress

Weichi Wu[1,2,3,6], Po Zhang[2,3,6], Donghai Wang[1,2,3], Xujia Wu[2,3], Qiulian Wu[1,2,3], Daqi Li[1,2,3], Tengfei Huang[1,2,3], Rui Wang[1,2,3], Huan Li[1,2,3], Hailong Mi[2,3], Suchet Taori[2,3], Fanen Yuan[2,3], Tingting Duan[2,3], Zhiye Chen[1,2,3], Huairui Yuan[2,3], Jeremy N. Rich[1,2,4,5]

[1]Lineberger Comprehensive Cancer Center, University of North Carolina, Chapel Hill, NC, USA

[2]UPMC Hillman Cancer Center, Pittsburgh, PA, USA

[3]Department of Medicine, University of Pittsburgh, Pittsburgh, PA, USA

[4]Department of Neurology, University of Pittsburgh, Pittsburgh, PA, USA

[5]Department of Neurology, University of North Carolina, Chapel Hill, NC, USA

[6]These authors contributed equally.

Corresponding author: Dr. Jeremy N. Rich, MD, MHS, MBA; Lineberger Comprehensive Cancer Center, University of North Carolina, Chapel Hill, NC, USA; Email: drjeremyrich@gmail.com

Conflict of Interest

The authors have declared that no conflict of interest exists.

Supplemental Material Content

Supplemental Figures 1-8

Supplemental Methods

Supplemental References

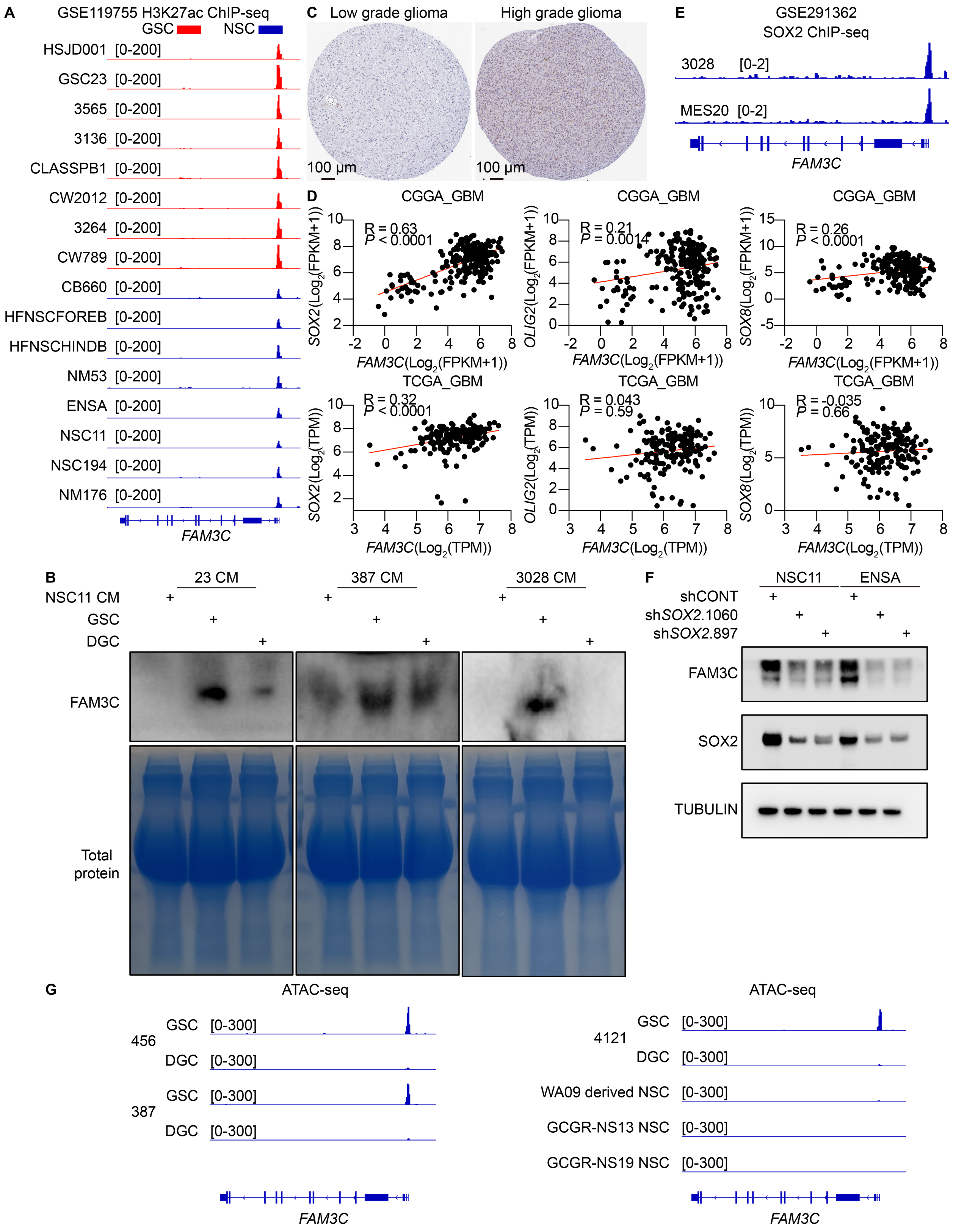
A
GSE119755 H3K27ac ChIP-seq
GSC
NSC
HSJD001 [0-200]
GSC23 [0-200]
3565 [0-200]
3136 [0-200]
CLASSPB1 [0-200]
CW2012 [0-200]
3264 [0-200]
CW789 [0-200]
CB660 [0-200]
HFNSCFOREB [0-200]
HFNSCHINDB [0-200]
NM53 [0-200]
ENSA [0-200]
NSC11 [0-200]
NSC194 [0-200]
NM176 [0-200]
FAM3C
C
Low grade glioma
High grade glioma
100 μm
100 μm
D
CGGA_GBM
R = 0.63
P < 0.0001
R = 0.21
P = 0.0014
R = 0.26
P < 0.0001
SOX2(Log2(FPKM+1))
OLIG2(Log2(FPKM+1))
SOX8(Log2(FPKM+1))
FAM3C(Log2(FPKM+1))
TCGA_GBM
R = 0.32
P < 0.0001
R = 0.043
P = 0.59
R = -0.035
P = 0.66
SOX2(Log2(TPM))
OLIG2(Log2(TPM))
SOX8(Log2(TPM))
FAM3C(Log2(TPM))
E
GSE291362
SOX2 ChIP-seq
3028 [0-2]
MES20 [0-2]
FAM3C
B
23 CM
387 CM
3028 CM
NSC11 CM
GSC
DGC
FAM3C
Total protein
F
NSC11
ENSA
shCONT
shSOX2.1060
shSOX2.897
FAM3C
SOX2
TUBULIN
G
ATAC-seq
456
387
GSC [0-300]
DGC [0-300]
4121
WA09 derived NSC [0-300]
GCGR-NS13 NSC [0-300]
GCGR-NS19 NSC [0-300]
FAM3C

Supplemental Figure 1. SOX2 drives FAM3C expression in GSCs.

(A) H3K27ac ChIP-seq tracks at FAM3C gene loci in GSCs and NSCs (GSE119755).

(B) Immunoblot analysis of secreted FAM3C levels in neurobasal medium used to culture GSCs, DGCs or NSCs for 48 hours. Sample loading was quantified by Ponceau S staining.

(C) Representative immunohistochemistry staining showing FAM3C expression in LGG and GBM. Scale bar, 100 μm.

(D) Pearson correlations between indicated genes expression in RNA-seq data of CGGA_GBM (*n* = 225 patients) and TCGA_GBM (*n* = 158 patients). Red lines show linear regression. Two-tailed Pearson correlation was used for statistical analysis.

(E) SOX2 ChIP-seq tracks at FAM3C gene loci in GSCs (GSC3028 and MES20) (GSE291362).

(F) Representative western blot analysis showing the effects of SOX2 knockdown on FAM3C protein levels in two NSC lines.

(G) ATAC-seq tracks showing the chromatin accessibility landscape at the FAM3C locus.

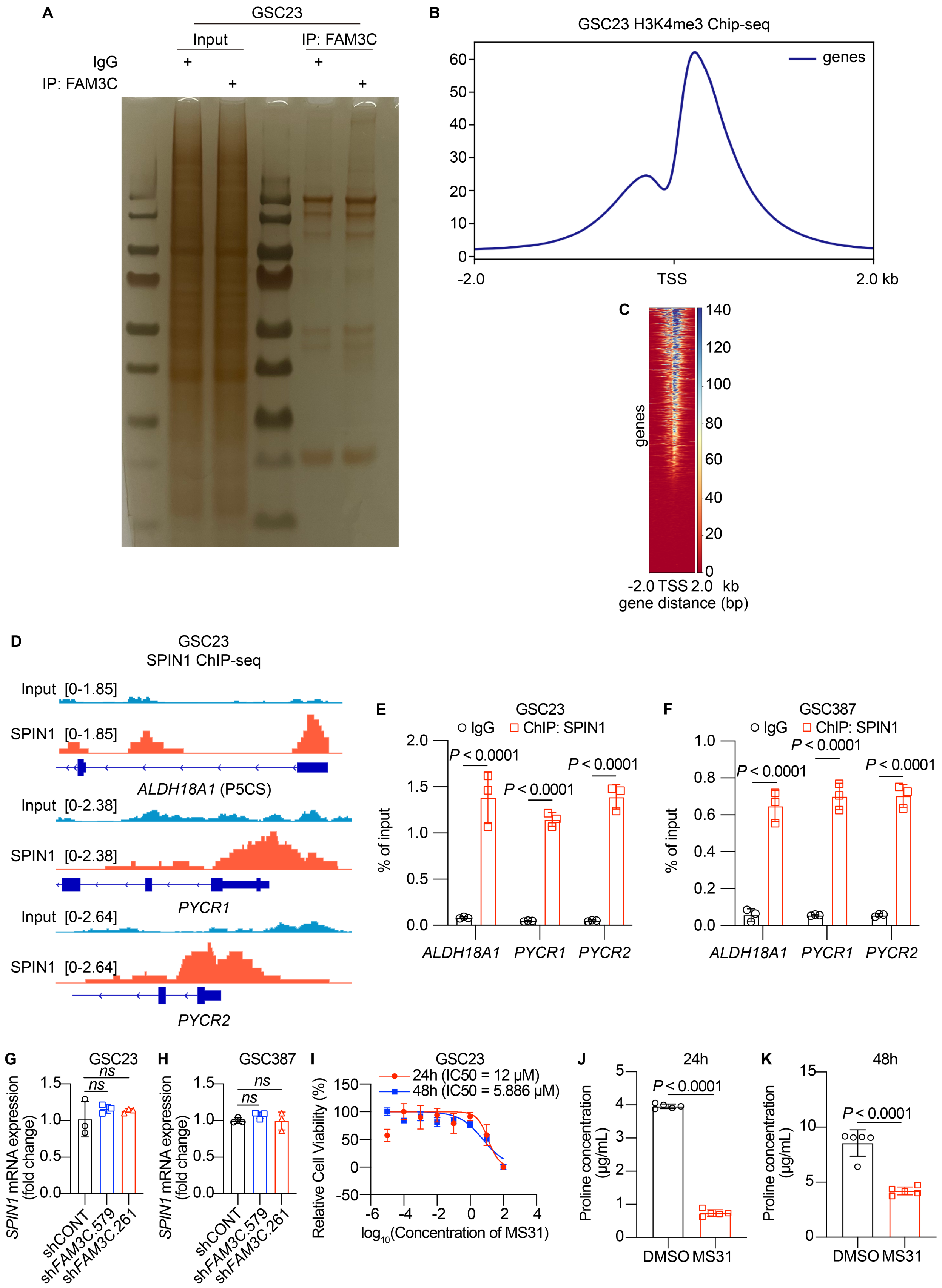
A
GSC23
Input
IP: FAM3C
IgG
IP: FAM3C
B
GSC23 H3K4me3 Chip-seq
genes
-2.0
TSS
2.0 kb
C
genes
140
120
100
80
60
40
20
0
-2.0 TSS 2.0 kb
gene distance (bp)
D
GSC23
SPIN1 ChIP-seq
Input [0-1.85]
SPIN1 [0-1.85]
ALDH18A1 (P5CS)
Input [0-2.38]
SPIN1 [0-2.38]
PYCR1
Input [0-2.64]
SPIN1 [0-2.64]
PYCR2
E
GSC23
IgG
ChIP: SPIN1
% of input
P < 0.0001
ALDH18A1
PYCR1
PYCR2
F
GSC387
IgG
ChIP: SPIN1
% of input
P < 0.0001
ALDH18A1
PYCR1
PYCR2
G
GSC23
SPIN1 mRNA expression (fold change)
ns
shCONT
shFAM3C.579
shFAM3C.261
H
GSC387
SPIN1 mRNA expression (fold change)
ns
shCONT
shFAM3C.579
shFAM3C.261
I
GSC23
24h (IC50 = 12 μM)
48h (IC50 = 5.886 μM)
Relative Cell Viability (%)
log10(Concentration of MS31)
J
24h
Proline concentration (μg/mL)
P < 0.0001
DMSO
MS31
K
48h
Proline concentration (μg/mL)
P < 0.0001
DMSO
MS31

Supplemental Figure 2. FAM3C interacts with SPIN1.

(A) IP-purified FAM3C protein complex from the whole lysates of GSC23 cells was analyzed by silver staining.

(B) The distribution of H3K4me3 peaks in GSC23 predominantly located near promoters, especially at transcription start sites (TSS).

(C) Heatmaps of H3K4me3 ChIP-seq signals in GSC23.

(D) Representative ChIP-seq tracks illustrating SPIN1 enrichment at the promoter regions of ALDH18A1 (P5CS), PYCR1, and PYCR2 in GSC23.

(E, F) ChIP-qPCR analysis of SPIN1 occupancy at the promoters of P5CS, PYCR1, and PYCR2 in (**E**) GSC23 and (**F**) GSC387 cells. IgG was used as a negative control. Data are presented as mean ± SD of three independent experiments. Statistical significance was determined by two-way ANOVA followed by Sidak's multiple comparisons test.

(G, H) RT-qPCR analysis of *SPIN1* in GSC23 (**G**) and GSC387 (**H**) with or without *FAM3C* KD (n = 3 independent experiments). Data are presented as mean ± SD. Statistical analysis was performed using one-way ANOVA followed by multiple comparison.

(I) Dose–response curves of MS31 in GSC23 (n = 5 independent experiments) for 24 hours or 48 hours. Data are presented as mean ± SD.

(J, K) The intracellular proline levels (n = 5 biologically independent samples) of GSC23 after treatment with MS31 for 24 (**J**) or 48 (**K**) hours with their respective IC_{50}. Data are presented as mean ± SD. Statistical analysis was performed using two-tailed unpaired t test.

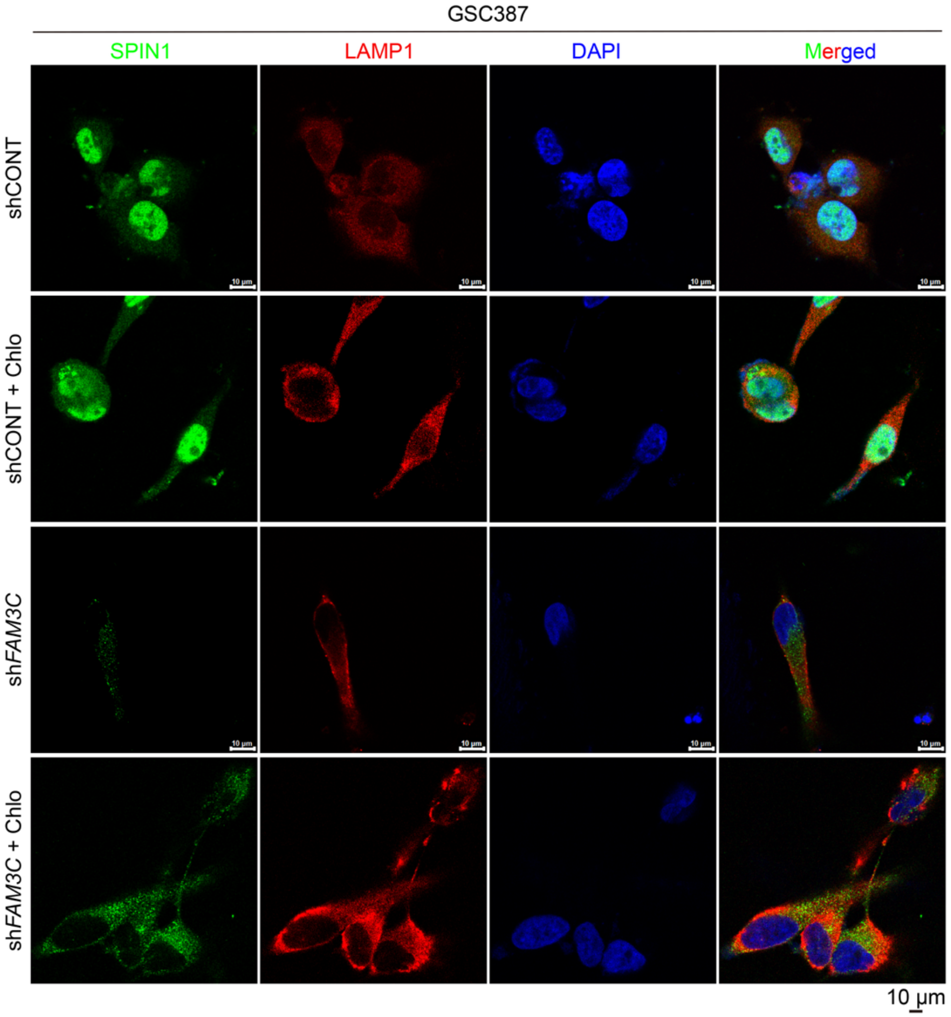

Supplemental Figure 3. FAM3C regulates SPIN1 stability and nuclear translocation to fuel proline biosynthesis. Representative images of SPIN1 (green) and a lysosomal marker, LAMP1 (red), in GSC387. Scale bar, 10 μm. DAPI (blue), 4,6-diamidino-2-phenylindole.

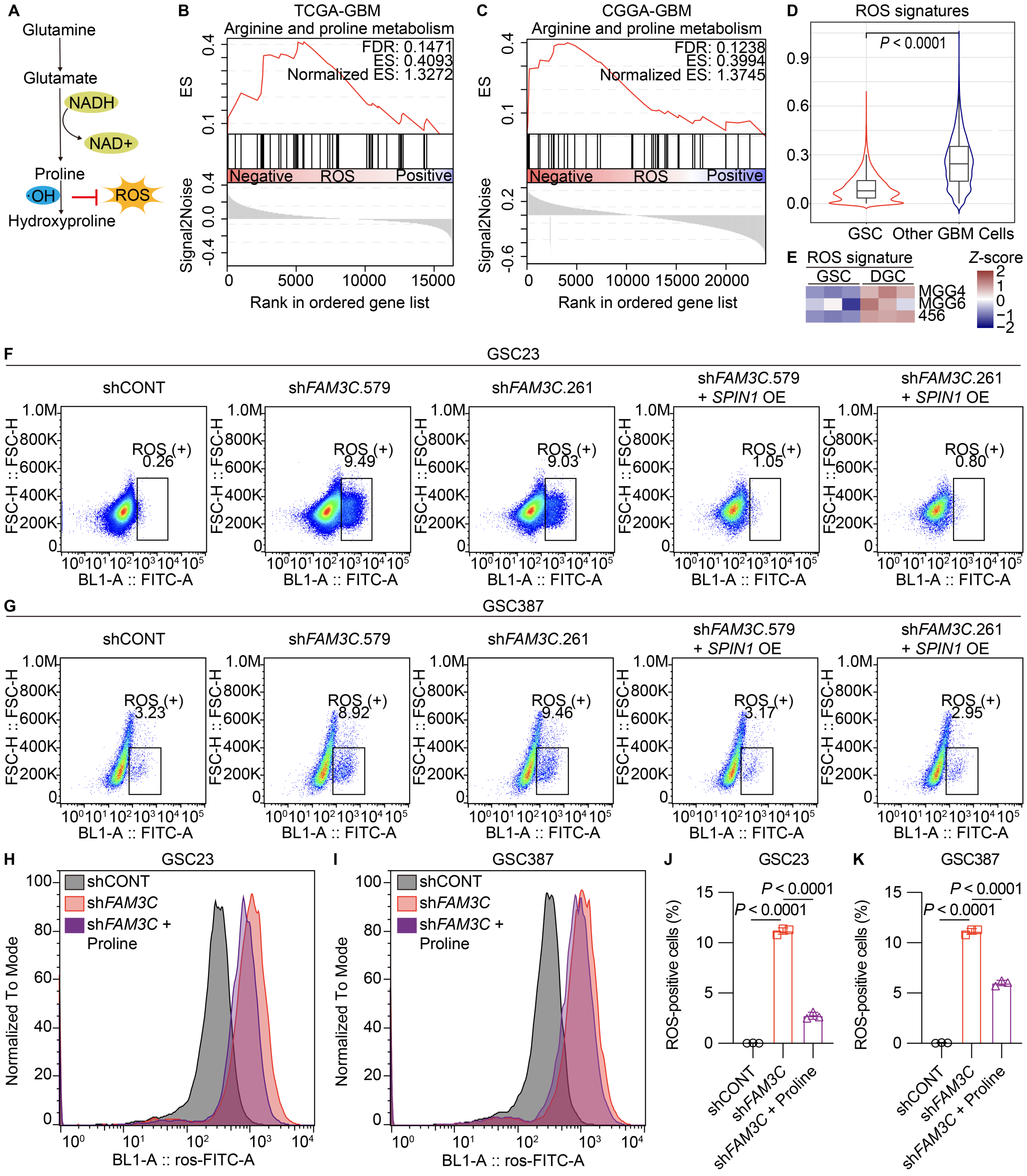
A
Glutamine
Glutamate
NADH
NAD+
Proline
OH
ROS
Hydroxyproline
B
TCGA-GBM
Arginine and proline metabolism
FDR: 0.1471
ES: 0.4093
Normalized ES: 1.3272
Negative ROS Positive
Rank in ordered gene list
C
CGGA-GBM
Arginine and proline metabolism
FDR: 0.1238
ES: 0.3994
Normalized ES: 1.3745
Negative ROS Positive
Rank in ordered gene list
D
ROS signatures
P < 0.0001
GSC Other GBM Cells
E
ROS signature
GSC DGC
MGG4
MGG6
456
Z-score
F
GSC23
shCONT
shFAM3C.579
shFAM3C.261
shFAM3C.579 + SPIN1 OE
shFAM3C.261 + SPIN1 OE
ROS (+) 0.26
ROS (+) 9.49
ROS (+) 9.03
ROS (+) 1.05
ROS (+) 0.80
FSC-H :: FSC-H
BL1-A :: FITC-A
G
GSC387
ROS (+) 3.23
ROS (+) 8.92
ROS (+) 9.46
ROS (+) 3.17
ROS (+) 2.95
H
GSC23
shCONT
shFAM3C
shFAM3C + Proline
Normalized To Mode
BL1-A :: ros-FITC-A
I
GSC387
J
GSC23
P < 0.0001
P < 0.0001
ROS-positive cells (%)
shCONT
shFAM3C
shFAM3C + Proline
K
GSC387

Supplemental Figure 4. FAM3C depletion induces loss of redox homeostasis.

(A) Overview of metabolic pathways mitigating oxidative stress.

(B, C) GSEA of Arginine and proline metabolism in TCGA-GBM (**B**) and CGGA-GBM (**C**) RNA-seq data, with samples divided into ROS-negative and ROS-positive groups based on the median ROS signature score from ssGSEA. Weighted Kolmogorov–Smirnov statistic test was used.

(D) Quantification of ROS signatures in scRNA-seq data of 28 early-passage GSC cultures derived from 24 patients and malignant cells from seven patients with GBM. *n* = 20,957 for all GSCs and *n* = 14,207 for all other tumor cells. Violin plots represent the overall distribution of data points. Box plots show median, upper and lower quartiles; whiskers depict 1.5 times the interquartile range. Statistical analysis was performed using two-tailed unpaired *t* test.

(E) Single-sample GSEA of metabolic gene sets from the KEGG database in three GSCs (MGG4, MGG6, 456) and matched DGCs. Each cell contains 3 replicates from GSE54791 and GSE247418.

(F, G) Representative gating strategy (*n* = 3 biologically independent samples per group) of ROS flow cytometric analysis in GSCs (GSC23 (**F**) and GSC387 (**G**)). The cutoff used to define positive (ROS (+)) and negative (ROS (-)) ROS signal is 10^3 on the logarithmic scale.

(H, I) Representative histogram plot of ROS flow cytometric analysis in GSC23 (**H**) and GSC387 (**I**) with or without *FAM3C* KD or in combination with *FAM3C* KD and proline supplement.

(J, K) Quantification of ROS-positive cells in GSC23 (**J**) and GSC387 (**K**) with or without *FAM3C* KD or in combination with *FAM3C* KD and proline supplement (*n* = 3 independent experiments). Statistical analysis was performed using one-way ANOVA followed by multiple comparison.

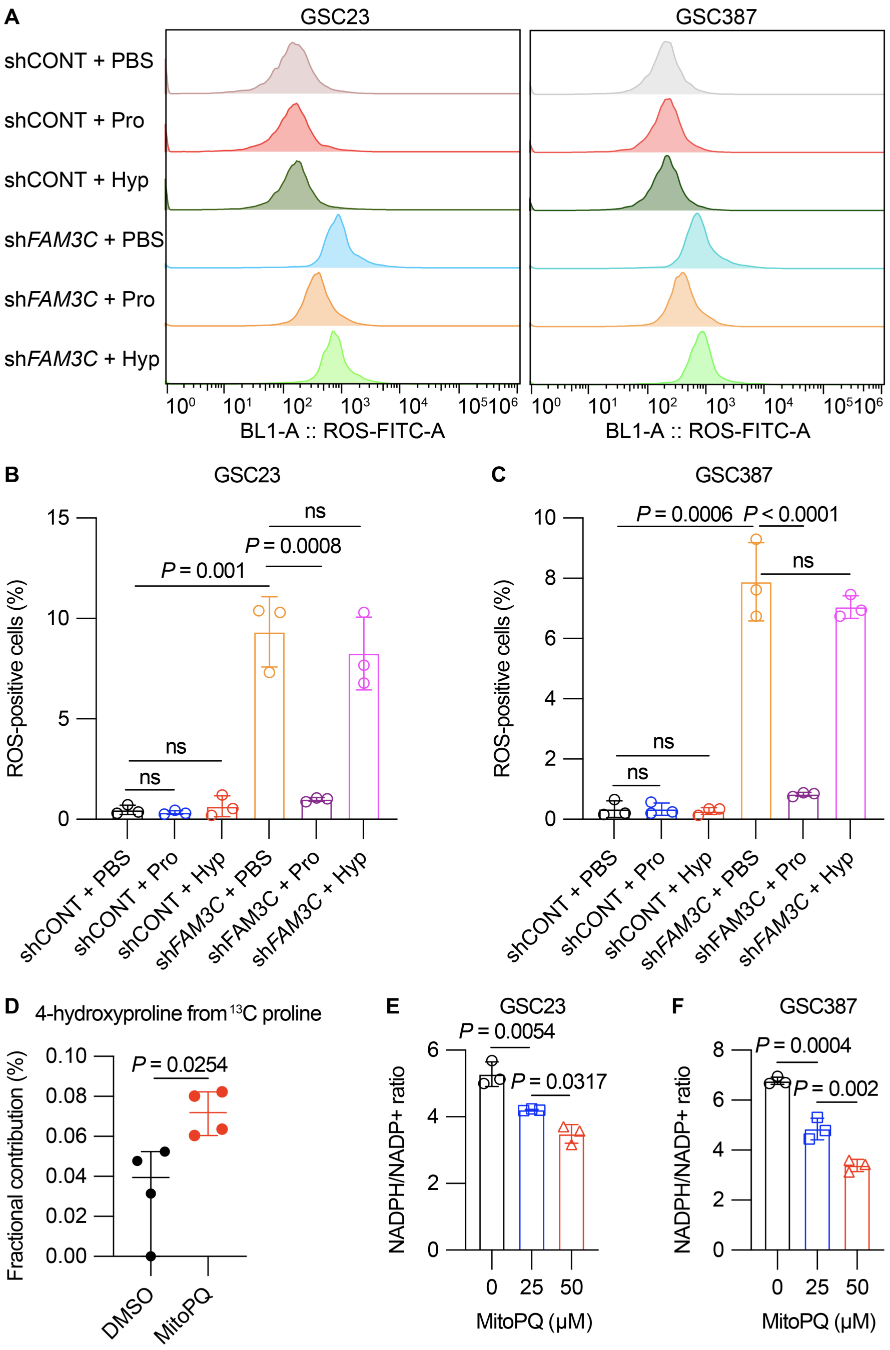
A
GSC23
GSC387
shCONT + PBS
shCONT + Pro
shCONT + Hyp
shFAM3C + PBS
shFAM3C + Pro
shFAM3C + Hyp
BL1-A :: ROS-FITC-A
B
GSC23
ROS-positive cells (%)
P = 0.001
P = 0.0008
ns
C
GSC387
P = 0.0006
P < 0.0001
D
4-hydroxyproline from 13C proline
Fractional contribution (%)
P = 0.0254
DMSO
MitoPQ
E
GSC23
NADPH/NADP+ ratio
P = 0.0054
P = 0.0317
MitoPQ (μM)
F
GSC387
P = 0.0004
P = 0.002

Supplemental Figure 5. Proline catabolism, but not hydroxyproline, is required for ROS suppression in GSCs.

(A) Representative flow cytometry plots of ROS-positive cells (FITC-A) in GSC23 and GSC387 cells expressing shCONT or shFAM3C, treated with PBS (control), proline (Pro), or hydroxyproline (Hyp) (200 μM) for 48 hours.

(B, C) Quantitative analysis of the percentage of ROS-positive cells in (**B**) GSC23 and (**C**) GSC387 (*n* = 3 biologically independent samples per group). Data are mean ± SD; statistical significance was determined by one-way ANOVA (P values as indicated; ns = not significant).

(D) MS analysis of labeled 4-hydroxyproline from [13C] proline tracing experiments for 4 h (*n* = 4 biologically independent samples per group) after oxidative stress induced by 50 μM MitoPQ for 48h in GSC23. Data are mean ± SD; statistical significance was determined by two-tailed unpaired t-test.

(E, F) Quantitative analysis of the NADPH/NADP+ ratio in (**E**) GSC23 and (**F**) GSC387 cells, treated with increasing concentrations of the mitochondrial superoxide inducer MitoPQ (0, 25, and 50 μM) for 48 hours (*n* = 3 biologically independent samples per group). Data are presented as mean ± SD; statistical significance was determined by one-way ANOVA.

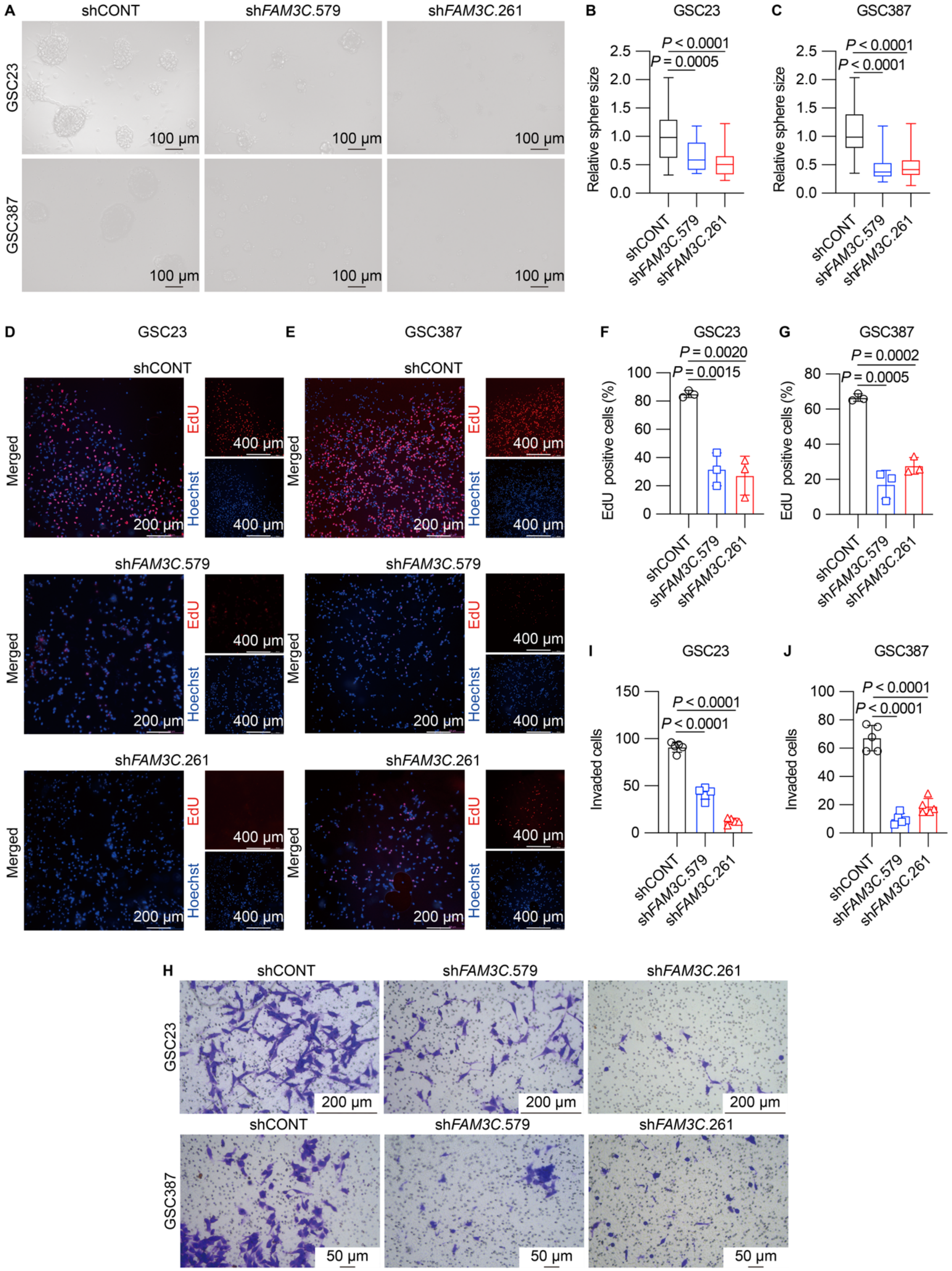
A
shCONT
shFAM3C.579
shFAM3C.261
GSC23
GSC387
100 μm
B
GSC23
P < 0.0001
P = 0.0005
Relative sphere size
C
GSC387
P < 0.0001
P < 0.0001
D
GSC23
E
GSC387
Merged
EdU
Hoechst
200 μm
400 μm
F
GSC23
P = 0.0020
P = 0.0015
EdU positive cells (%)
G
GSC387
P = 0.0002
P = 0.0005
I
GSC23
P < 0.0001
P < 0.0001
Invaded cells
J
GSC387
P < 0.0001
P < 0.0001
H
50 μm

Supplemental Figure 6. FAM3C promotes GSC proliferation and predicts poor prognosis.

(A) Representative images of tumorspheres of GSC23 and GSC387 cells with or without FAM3C KD. Scale bar, 100 μm.

(B, C) Quantification of sphere formation (GSC23 (**B**), *n* = 38 (shCONT), 23 (sh*FAM3C*.579), 33 (sh*FAM3C*.261); GSC387 (**C**), *n* = 29 (shCONT), 66 (sh*FAM3C*.579), 50 (sh*FAM3C*.261) in GSCs with or without *FAM3C* KD.

(D, E) Representative images of EdU incorporation in GSC23 (**D**) and GSC387 (**E**) with or without *FAM3C* KD. Scale bar, 200 μm for merged channels, 400 μm for individual channels.

(F, G) Quantification of EdU incorporation (*n* = 3 randomly selected fields per group) in GSCs (GSC23 (**F**) and GSC387 (**G**)) with or without *FAM3C* KD.

(H) Representative images of invasion of GSC23 and GSC387 cells with or without FAM3C KD. Scale bars, 200 μm for GSC23, and 50 μm for GSC387.

(I, J) Quantification of invasion assay (*n* = 5 randomly selected fields per group) in GSCs (GSC23 (**I**) and GSC387 (**J**)) with or without *FAM3C* KD.

In **B** and **C**, Boxes represent data within the 25-to-75 percentiles. Whiskers depict the range of all data points. Horizontal lines within boxes represent mean values. In **F**, **G**, **I** and **J**, data are presented as mean ± SD. One-way ANOVA followed by multiple comparison for **B, C, F, G**, **I** and **J**.

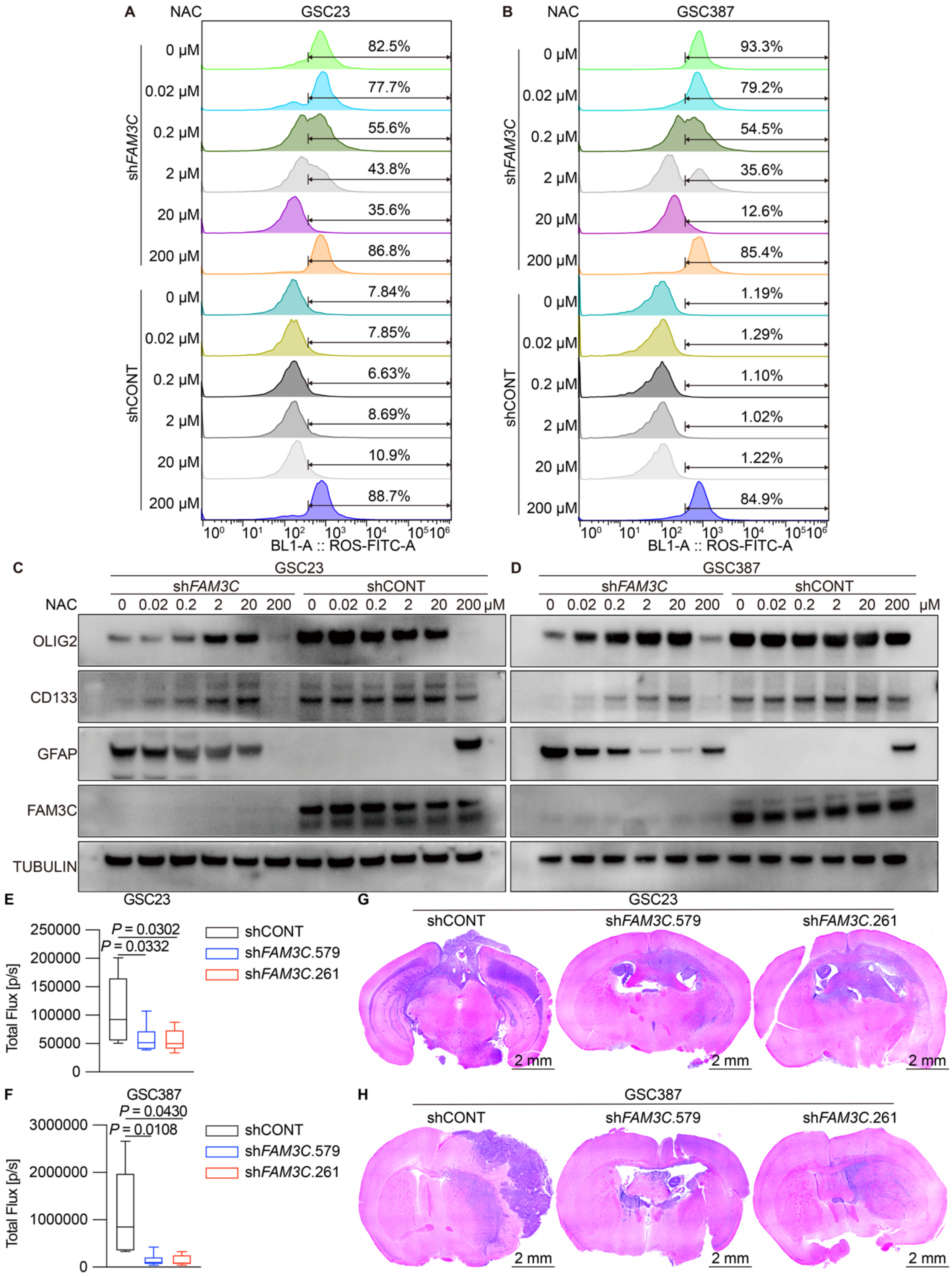

A
NAC
GSC23
shFAM3C
shCONT
0 μM
0.02 μM
0.2 μM
2 μM
20 μM
200 μM
82.5%
77.7%
55.6%
43.8%
35.6%
86.8%
7.84%
7.85%
6.63%
8.69%
10.9%
88.7%
BL1-A :: ROS-FITC-A
B
GSC387
93.3%
79.2%
54.5%
35.6%
12.6%
85.4%
1.19%
1.29%
1.10%
1.02%
1.22%
84.9%
C
D
OLIG2
CD133
GFAP
FAM3C
TUBULIN
E
F
Total Flux [p/s]
P = 0.0302
P = 0.0332
P = 0.0430
P = 0.0108
shCONT
shFAM3C.579
shFAM3C.261
G
H
2 mm

Supplemental Figure 7. FAM3C maintains GSC stemness by reducing ROS production and promotes in vivo proliferation.

(A, B) Representative flow cytometry plots measuring ROS levels in (**A**) GSC23 and (**B**) GSC387 cells. GSCs expressing shCONT or sh*FAM3C* were treated with increasing concentrations of N-acetylcysteine (NAC; 0, 0.02, 0.2, 2, 20, and 200 μM) for 48 hours; percentages represent ROS-positive populations.

(C, D) Western blot analysis of stemness markers (OLIG2, CD133) and the differentiation marker GFAP in (**C**) GSC23 and (**D**) GSC387 cells under the same treatment conditions.

(E, F) Quantification of total flux of in vivo bioluminescence images of tumor-bearing mice derived from GSC23 (**E**) and GSC387 (**F**) with or without *FAM3C* KD on Day 21. Boxes represent data within the 25-to-75 percentiles. Whiskers depict the range of all data points. Horizontal lines within boxes represent mean values. One-way ANOVA followed by multiple comparison was used for statistical analysis.

(G, H) Representative images of hematoxylin and eosin (H&E)-stained brain sections (scale bar, 2 mm) of tumor-bearing mice derived from GSC23 (**G**) and GSC387 (**H**) with or without *FAM3C* KD.

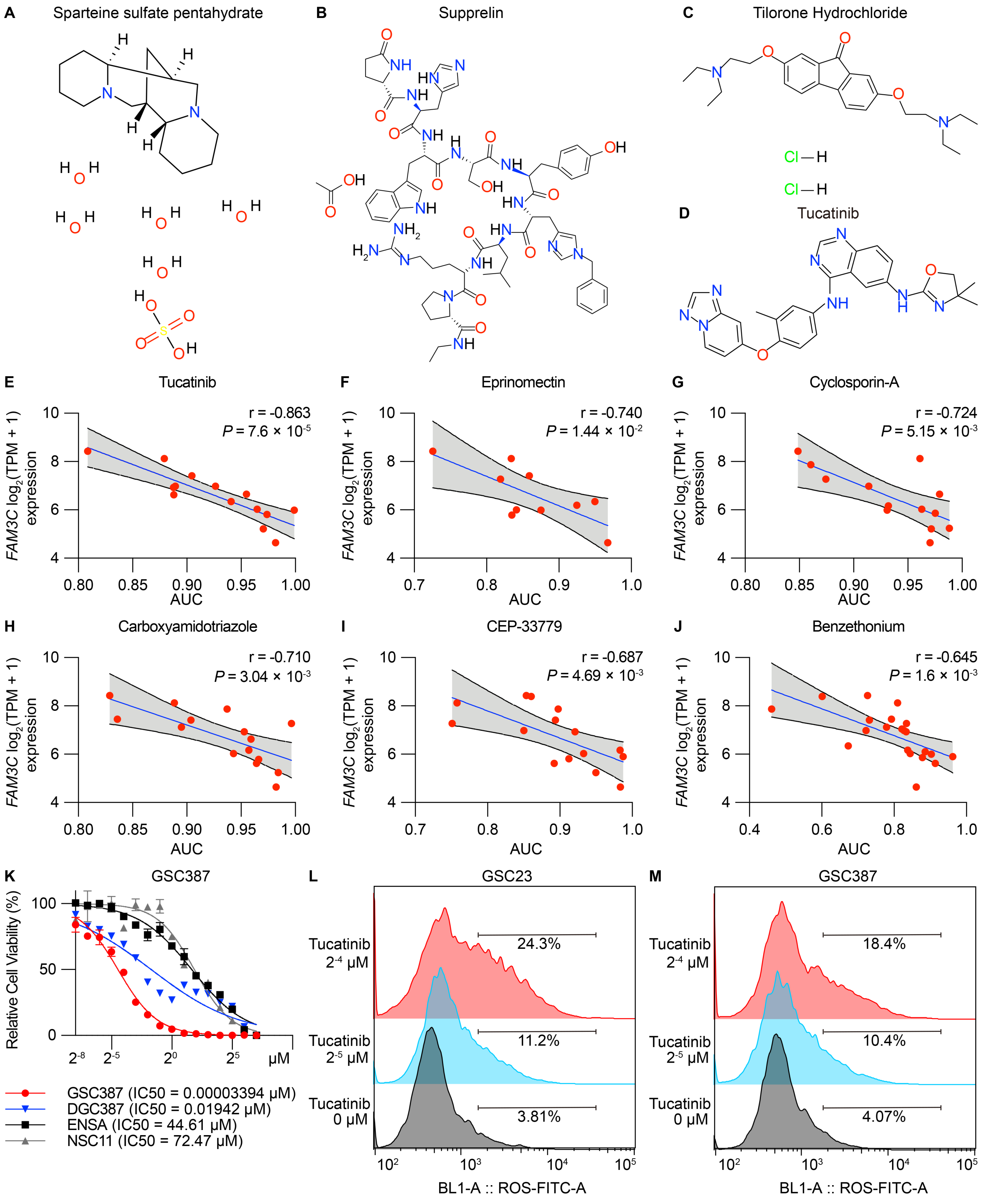

A
Sparteine sulfate pentahydrate
B
Supprelin
C
Tilorone Hydrochloride
D
Tucatinib
E
Tucatinib
r = -0.863
P = 7.6 × 10-5
F
Eprinomectin
r = -0.740
P = 1.44 × 10-2
G
Cyclosporin-A
r = -0.724
P = 5.15 × 10-3
H
Carboxyamidotriazole
r = -0.710
P = 3.04 × 10-3
I
CEP-33779
r = -0.687
P = 4.69 × 10-3
J
Benzethonium
r = -0.645
P = 1.6 × 10-3
FAM3C log2(TPM + 1) expression
AUC
K
GSC387
Relative Cell Viability (%)
μM
GSC387 (IC50 = 0.00003394 μM)
DGC387 (IC50 = 0.01942 μM)
ENSA (IC50 = 44.61 μM)
NSC11 (IC50 = 72.47 μM)
L
GSC23
M
GSC387
Tucatinib 2-4 μM
Tucatinib 2-5 μM
Tucatinib 0 μM
24.3%
11.2%
3.81%
18.4%
10.4%
4.07%
BL1-A :: ROS-FITC-A

Supplemental Figure 8. Tucatinib disrupts the interaction between FAM3C and SPIN1.

(A-D) Structure of the four most highly rated molecules in virtual screening analyses, including Sparteine sulfate pentahydrate (**A**), Supprelin (**B**), Tilorone Hydrochloride (**C**) and Tucatinib (**D**).

(E-J) Scatter plots showing correlation between FAM3C expression levels and AUC values of Tucatinib (**E**), Eprinomectin (**F**), Cyclosporin-A (**G**), Carboxyamidotriazole (**H**), CEP-33779 (**I**) or Benzethonium (**J**) in brain cancer cell lines. Pearson correlation coefficients and P values are shown.

(K) Dose–response curves of Tucatinib in GSC387, DGC387 and NSCs (NSC11 and ENSA) (*n* = 3 independent experiments). Data are presented as mean ± SD.

(L) The intracellular proline levels (*n* = 5 biologically independent samples) of GSC387 after treatment with gradually increasing concentrations of Tucatinib (0 μM, 2^{-5} μM, 2^{-4} μM) for 6 hours. Data are presented as mean ± SD. Statistical analysis was performed using two-tailed unpaired *t* test.

(M, N) Representative histogram plot of ROS flow cytometric analysis in GSC23 (**M**) and GSC387 (**N**) after treatment with gradually increasing concentrations of Tucatinib (0 μM, 2^{-5} μM, 2^{-4} μM) for 6 hours. The cutoff used to define positive (ROS (+)) and negative (ROS (-)) ROS signal is 10^3 on the logarithmic scale.

Supplemental methods

This study complies with all relevant ethical standards and has been approved by the Ethics Committee and Institutional Review Board at the University of Pittsburgh and University of North Carolina, Chapel Hill.

GSC dataset interrogation

We obtained RNA-seq data for three pairs of GSCs and DGCs from GSE54791 (1), which we subsequently analyzed using the limma package. To determine proline metabolism activity in GSCs, we employed GSEA, referencing the KEGG_ARGININE_AND_PROLINE_METABOLISM pathway. Additionally, RNA-seq data encompassing 44 GSC samples and 9 NSC samples, sourced from GSE119834 (2), also underwent analysis with the limma package. ChIP-seq data were retrieved from GSE54047, GSE119755, GSE129438 and GSE185954. For visualizing the ChIP-seq data peaks, we utilized Integrative Genomics Viewer (IGV).

GSCs derivation and cell culture

GSCs isolated from patients were acquired and cultured following established protocols (3). The GSC23 sample originated from a biopsy taken from a 63-year-old man's recurrent GBM (3). This sample was kindly provided by E. Sulman of NYU Langone Health (3). The GSC387 cell line originated from a GBM tumor in a 76-year-old female patient (3). GSC3028 was established using a recurrent glioblastoma (GBM) sample from a 65-year-old female (3). To reduce potential artifacts from in vitro cell culture, patient-derived xenografts were grown to provide a consistent supply of GSCs. Additionally, the NSC11 line (hNSC11, Alstem) was

developed from human induced pluripotent stem cells (iPSCs). Separately, ENSA (ENStem-A, Millipore) refers to human embryonic stem cell-derived neural progenitors. All GSC and NSC lines were maintained at 37 °C under 20% oxygen and 5% carbon dioxide. The culture medium consisted of Neurobasal medium (Gibco, 21103049) fortified with B27 without vitamin A (Gibco, 12587010), 20 ng/ml of recombinant human EGF (R&D Systems, 236-EG-01M), and 20 ng/ml of recombinant human bFGF (R&D Systems, 4114-TC-01M). Additionally, sodium pyruvate (Gibco, 11360070), GlutaMAX (Gibco, 35050061) and streptomycin–penicillin (Gibco, 15140122) were included. Matched serum-derived glioblastoma cells (DGCs) were kept in DMEM (Gibco, 11995065) containing 10% FBS (Gibco, 26140079) to preserve their differentiated state. HEK293T cells, obtained from ATCC (CRL-3216), were cultured in DMEM that also included 10% FBS. To verify the identity of the tumor models utilized in this research, short tandem repeat (STR) analyses were carried out on an annual basis. Furthermore, mycoplasma testing was conducted at least yearly to confirm the cultures remained free from contamination. All experimental protocols adhered to relevant regulatory guidelines.

Lentivirus production

For lentivirus production, the shRNA plasmids utilized in this study were acquired from Sigma. Details concerning the shRNA sequences can be found in Supplemental Table 1. We co-transfected transfer plasmids with psPax2 (Addgene, 12260) and pMD2G (Addgene, 12259) into HEK293T cells, employing PEI (Polysciences, 24765-100) as the transfection reagent. Lentiviruses were subsequently harvested on days 1, 2, and 3 following transfections.

Immunoblotting

Immunoblotting was carried out following standard protocols. Detailed information about the antibodies used can be found in Supplementary Table 2.

Liquid Chromatography-Mass Spectrometry (LC-MS) for amino acid profiling

The samples were extracted for amino acids using a methanol: chloroform extraction. An aliquot of the samples was resuspended in the 20 mM HCl and derivatized using the AccQ-Tag reagent from waters. The samples were analyzed using an LC-MS based on MRM (Multiple Reaction Monitoring). The Shimadzu UPLC Nexera II interfaced with a Sciex QTRAP 6500+ mass spectrometer equipped with a TurboIonSpray (TIS) electrospray ion source. The column used was ACCQ-TAG Ultra C18 1.7µm, 2.1x100 mm (Waters) and was run at a flow rate of at 0.4 mL/min at 45°C. The gradient of the mobile phases A (0.1% formic acid in water) and B (0.1% formic acid in acetonitrile) was as follows: 4% B to 30% B in 7 min, then ramped up to 90% B in 0.5 min, held at 90% for 2.5 min, back down to 4% B in 0.5 min. Samples were further diluted to fit within the linear dynamic range for the quantification using the LC-MS method. An external standard curve was run using known concentrations of Ala, Arg, Asn, Asp, Cys2, GABA, Gln, Glu, His, HyPro, Ile, Leu, Lys, Met, Orn, Phe, Pro, Ser, Tau, Thr, Trp, Tyr, and Val.

The standard curves were used to calculate the concentration of all amino acids in the samples from the peak area detected. The results in µg/cell pellet of each compound are summarized in Supplementary Table 3. Only detected amino acids were reported.

RNA sequencing and data analysis

Total RNA isolation and library preparation for sequencing were performed by Novogene, utilizing the Illumina TruSeq Stranded Total RNA Library Prep Kit. The prepared libraries underwent paired-end sequencing, generating 150-bp reads. Raw FASTQ data were then subjected to quality trimming using Trim Galore. Transcript alignment to the human reference genome (hg38) was carried out with HISAT2. Subsequent processing, including sorting, indexing, and format conversion from SAM files, was achieved with SAMtools. For gene quantification and differential expression analysis, featureCounts and DESeq2 were employed, respectively. Differentially expressed genes (DEGs) were defined by an absolute fold change exceeding 1.5 and an adjusted *P*-value less than 0.05. Gene Ontology (GO) enrichment and gene set enrichment analysis (GSEA) were conducted using the clusterProfiler package. Finally, single-sample GSEA (ssGSEA), implemented via the GSVA package's ssGSEA method in R, was used to evaluate individual pathway activities. The gene counts of all samples are summarized in Supplementary Table 4.

RNA extraction and quantitative real-time PCR

For gene expression analysis, total RNA was extracted using both TRIzol Reagent (Life Technologies, 15596018) and the Direct-zol RNA Miniprep Kit (Zymo Research, R2052). Complementary DNA (cDNA) was then synthesized via reverse transcription with the High-Capacity cDNA Reverse Transcription Kit (Thermo Fisher Scientific, 4368814). Quantitative real-time polymerase chain reaction (qPCR) experiments were executed on a CFX96 Touch Real-Time PCR Detection System (Bio-Rad) employing SYBR Green Master Mix (Thermo Fisher Scientific, 4309155). Transcript levels were normalized against ACTB expression. A

comprehensive list of the primers used for this investigation can be found in Supplementary Table 5.

Public glioma patient datasets

The mRNA expression of FAM3C was investigated using publicly accessible glioma databases from GlioVis (http://gliovis.bioinfo.cnio.es). For the subsequent survival analysis, only IDH-WT glioma samples were considered. Patient survival data were then assessed via Kaplan–Meier curves and evaluated for statistical significance using the log-rank test.

Single-cell RNA sequencing (scRNA-seq) reanalysis

For the GSE84465 dataset, initial steps included data normalization, followed by differential expression analysis across all cells. We then conducted principal component analysis (PCA), which preceded clustering and UMAP-based dimensionality reduction. Cell annotation relied on established markers for GSCs and DGCs as defined by Martina et al. (4), with the CellMarker database (http://xteam.xbio.top/CellMarker/#) serving as a reference for identifying other cellular phenotypes.

For the scRNA-seq dataset used in Fig 6D, we utilized scRNA-seq data comprising 65,655 cells from 28 early-passage GSC cultures, originating from 24 distinct patients. Additionally, 14,207 malignant tumor cells sourced from seven Glioblastoma multiforme (GBM) specimens were included. The initial cellular annotations and PCA coordinates were adopted directly from the original datasets and associated code.

Our initial step involved identifying differentially expressed genes (DEGs) between in vitro GSC cultures and in vivo tumor cells. This was achieved using the FindMarkers function within the Seurat package in R, with significance defined as an absolute Log2 fold change greater than 2 and an adjusted P-value less than 0.05.

To distinguish GSCs from DGCs within the 14,207 malignant tumor cells, a logistic regression classifier was developed as previously detailed. Specifically, this classifier was trained on a designated training set. Optimal hyperparameters were determined through five-fold cross-validation using the caret package in R, leveraging the identified DEGs. We robustly evaluated the model's accuracy and stability by repeating an 80–20 stratified random train–test split 30 times. The model demonstrated high accuracy and robustness, and the top-performing model (based on highest test accuracy) was selected to predict the classification across the entire dataset. Consequently, 13,226 correctly classified tumor cells were re-designated as DGCs, while 981 misclassified tumor cells were re-assigned as GSCs.

The inferred activity related to mechanical stimulus sensing was determined using AUCell (version 1.20.2), based on the Gene Ontology Biological Process (GOBP) term "Detection of mechanical stimulus." Scores were then normalized to a 0–1 range by subtracting the minimum value and dividing by the full range. For statistical comparisons, a two-tailed unpaired t-test was employed.

ChIP–qPCR

ChIP experiments were conducted utilizing the Magna ChIP A/G Chromatin Immunoprecipitation Kit (Millipore, catalog no. 17-10085). Cellular samples underwent cross-linking with 1% formaldehyde for 10 minutes, with the reaction subsequently quenched by the addition of 0.125 M glycine. Following this, nuclei were isolated, lysed, and sonicated to achieve chromatin fragmentation into segments ranging from 200 to 500 base pairs. The resultant sheared chromatin was then diluted and subjected to overnight incubation with either 5 μg of the target-specific antibody or IgG. This was followed by capture using magnetic protein A/G beads (Thermo Fisher Scientific, catalog no. 26162). A series of stringent washing steps were performed, after which chromatin was eluted, cross-links were reversed, and the ChIP DNA was purified for subsequent PCR analysis. Comprehensive information regarding the primers and antibodies employed in this study can be found in Supplementary Table 2 and 5.

ChIP-Seq analysis

ChIP-Seq was performed as previously described with a few modifications (5). For chromatin Immunoprecipitation sequencing analysis (ChIP-Seq), GSC cells were cross-linked with 1% paraformaldehyde for 10 minutes, followed by quenching with glycine for 5 minutes at room temperature. After cell lysis, fixed samples were sonicated to generate DNA fragments ranging from 200 to 600 bp. Immunoprecipitation was performed using a SPIN1 antibody (Proteintech, 12105-1-AP) and Magna ChIP Protein A/G Magnetic Beads (Sigma-Aldrich, 16-663). 1 to 10 ng of immunoprecipitated DNA and the NEBNext Ultra DNA Library Prep Kit for Illumina were used for library preparation, and the samples were subjected to sequencing on the NovaSeq X Plus. Raw ChIP-seq reads were aligned to the human genome (hg38) using Bowtie2 (version 2.5.3) with default settings. Quality control was performed using FastQC (version 0.12.0), and adapter trimming was done with

Trimmomatic (version 0.39). Read coverage was calculated using deepTools (version 3.5.5) and visualized in the IGV software.

MS analysis

Liquid chromatography–MS/MS (LC–MS/MS) analysis was conducted at the University of Pittsburgh's Health Sciences Mass Spectrometry Core. Peptide abundances were employed for protein quantification. Log2 fold changes (Log2FC) and corresponding p-values for each sample were recomputed using the DEP package in R.

Co-immunoprecipitation analysis

In short, cells were lysed using IP buffer (Thermo Fisher Scientific, 87788) that contained 1 mM NaF, 1 mM Na_3VO_4, 1 mM PMSF, and a protease-inhibitor cocktail (Thermo Fisher Scientific, A32953). After removing cellular debris via centrifugation, the resulting lysates were pre-cleared for two hours at 4 °C with IgG antibody (from the same species as the immunoprecipitation antibody) and protein G beads (Thermo Fisher Scientific, 10003D) to minimize non-specific binding. Subsequently, the clarified lysates were incubated overnight at 4 °C with either the designated primary antibody and protein G beads, or anti-FLAG M2 beads (Sigma-Aldrich, M8823). The immunoprecipitate was then washed three times with IP buffer, boiled, and analyzed via immunoblotting following standard procedures.

Proline tracing and mass spectrometry analysis of hydroxyproline

680 µM of the amino acid L-Proline-^{13}C (MedChemExpress, HY-Y0252S3) was added into the medium. Cells were traced for 4 h, collected and then subjected to MS analysis of 4-

hydroxyproline. The raw data were integrated manually using MassLynx software (v4.1, Waters, Milford, MA, USA).

Cytoplasm and nucleus fractionation

Cytoplasmic and nuclear components were isolated using the Nuclear Extract Kit (Active Motif, 40010), following the manufacturer's instructions. Subsequently, whole-cell, cytoplasmic, and nuclear extracts from GSCs were subjected to immunoblotting with specific antibodies.

Proline quantification

Proline (Pro) concentrations were determined using the Proline Colorimetric Assay Kit (Elabscience, E-BC-K177-S), following the manufacturer's instructions.

Glycine quantification

Glycine (Gly) concentrations were determined using the Glycine Assay Kit (Cell Biolabs, MET-5070), following the manufacturer's instructions.

NADPH/NADP+ quantification

NADPH/NADP+ ratio was determined using the Bioluminescent Assays to Detect NAD(P)+ and NAD(P)H Levels in Cells Kit (Promega, G9081), following the manufacturer's instructions.

Immunofluorescence analysis

For immunofluorescence, cells were cultivated on Matrigel-coated coverslips. They were then fixed with 4% paraformaldehyde (PFA) for 15 minutes, followed by permeabilization in

0.1% Triton X-100 in PBS for an additional 15 minutes. After three PBS washes, the cells were blocked for one hour in PBS buffer containing 5% goat serum. Relevant primary antibodies, diluted in this blocking buffer, were applied and incubated overnight at 4°C. This was followed by a one-hour incubation with secondary antibodies at room temperature, adhering to the manufacturer's guidelines.

ROS detection and flow cytometry analysis

ROS levels in GSCs were quantified using the Total ROS Assay Kit 520 nm (ThermoFisher, 88-5930-74), following the manufacturer's protocol. Briefly, a 500X stock solution of the ROS Assay Stain was prepared by introducing 40 μL of dimethyl sulfoxide (DMSO) into the ROS Assay Stain Concentrate vial and mixing thoroughly. For cell labeling, this 500X stock solution was employed at a 1X working concentration. It could be directly added to cells within their culture medium to achieve a final 1X concentration by incorporating 2 μL of the 500X stock solution per 1 mL of cell suspension, ensuring proper mixing. Alternatively, the 500X ROS Assay Stain stock solution could be diluted to 1X using the provided ROS Assay Buffer. Each sample necessitated 100 μL of the 1X ROS Assay Stain Solution for cell resuspension. Cells were then incubated for 60 minutes in a 37°C incubator under 5% CO_2. Subsequently, cells were treated with as experiment design to induce ROS production. Flow cytometry analysis was performed using the LSR Fortessa (BD) system, and data were processed with FlowJo software (v.10).

Neurosphere formation and proliferation assay

We assessed neurosphere formation capacity using in vitro extreme limiting dilution assays. Cells were seeded at declining densities (50, 25, 12, 6, 3, and 1 cell per well) into a 96-well plate. The number and existence of neurospheres in each well were then evaluated between 7 and 14 days after plating. Data analysis for these assays was conducted utilizing the software found at http://bioinf.wehi.edu.au/software/elda. Additionally, cell viability was measured at specific time points using CellTiter-Glo (Promega).

Click-iT EdU incorporation assay

Cellular proliferation rates were determined utilizing the Click-iT EdU Cell Proliferation Kit (Thermo Fisher Scientific, C10339). Cells were treated with 10 μM EdU for a 2-hour incubation period. Subsequently, three randomly selected microscopic fields were imaged for each experimental condition. The proportion of proliferating cells was then calculated by normalizing the count of EdU-positive cells to the total number of DAPI-stained nuclei, with all image analysis conducted using ImageJ software.

Invasion assay

Prior to experimentation, all GSCs underwent a 24-hour starvation period in B27-free medium. Then we seeded 1×10^5 GSCs in B27-free medium into 24-well inserts (8.0 μm). Complete medium was then added to the receiver wells. After a 24-hour incubation, migrated macrophages were fixed using 4% formaldehyde. We then stained these cells with crystal violet and quantified them under a microscope.

Xenografts

All murine experiments adhered to the guidelines set by the Institutional Animal Care and Use Committee of the University of Pittsburgh (protocol 21049014). Mice were kept in specific-pathogen-free environments with ambient temperatures between 20–26 °C, humidity levels of 30–70%, and a 12-hour light/dark cycle prior to experimentation. Both male and female mice were included in these studies.

For intracranial xenograft procedures, 4–6-week-old healthy NSG mice (NOD.Cg-*Prkdc*^{scid} *Il2rg*^{tm1Wjl}/SzJ, strain 005557, Jackson Laboratory) were randomly chosen. Each mouse received an intracranial injection of 10,000 GSCs into the right cerebral cortex at a depth of 3.5 mm, following established protocols. A veterinarian oversaw housing conditions and animal well-being. Mice were monitored until they exhibited neurological symptoms—such as hunched posture, altered gait, lethargy, or weight loss—at which point they were humanely euthanized. Their brains were then collected, preserved in 4% paraformaldehyde, and prepared for paraffin-embedded sectioning. Histological analysis was conducted on these sections using H&E staining. Mouse survival data was analyzed using GraphPad Prism software, with statistical significance determined by the log-rank test.

Mice that received intracranial implants of firefly luciferase-labeled GSCs underwent bioluminescence imaging for monitoring. Animals were intraperitoneally administered D-luciferin (50 mg/kg, Promega, P1042) and anesthetized with isoflurane for the imaging sessions. Bioluminescent images were captured utilizing an IVIS imaging system (PerkinElmer).

Virtual screening docking

The crystal structure of FAM3C (PDB code: 5LC2) and SPIN1 (PDB code: 2NS2) was obtained from the Protein Data Bank. Prior to molecular docking, the protein was preprocessed using the Protein Preparation Wizard module within the Schrödinger software package. This involved removing crystal water molecules, adding hydrogen atoms, and assigning charges and residue protonation states (at pH 7.0 ± 2.0) using the OPLS_4 force field. The structure then underwent energy optimization. For docking simulations, the highest-scoring site identified by SiteMap was used to define the grid box center, ensuring the entire target protein's active site was enclosed within the grid. All other grid box settings were maintained at their default values. Virtual screening was performed against the CNPD database of natural products. Small molecules from this database were preprocessed using the LigPrep module. Docking scores below -6 were considered indicative of effective binding.

Drug therapeutic efficacy prediction of FAM3C

Drug therapeutic efficacy was predicted through an analysis combining drug screening data from the CTRP (version 2) with corresponding mRNA expression profiles of cell lines from the Cancer Cell Line Encyclopedia. For each tested compound, we determined the Pearson correlation coefficient and its associated two-tailed *P*-value, assessing the relationship between FAM3C expression levels and the AUC values across 133 brain cancer cell lines. The list of brain cancer cell lines and the comprehensive correlation outcomes can be found in Supplementary Table 6.

Cellular Thermal Shift Assay (CETSA)

The direct interaction of Tucatinib with FAM3C protein was explored using the CETSA, as detailed in a prior publication (6). Approximately 10^7 HEK293T cells, engineered to overexpress FAM3C, were pre-incubated with 200 nM Tucatinib for 6 hours before starting the CETSA. Cells from each condition were harvested, washed once with chilled PBS, and reconstituted in 1.5 mL of PBS containing protease inhibitors. This cell suspension was portioned into seven 100 μL aliquots within 0.2-mL PCR tubes. To facilitate protein denaturation, samples were heat-shocked for 3 minutes at specified temperatures using a Bio-Rad T100 Thermal Cycler, followed by immediate 3-minute cooling to ambient temperature. Cell lysis was achieved by performing three rapid freeze-thaw cycles using dry ice and a thermal cycler set to 25°C. Lysates were then clarified by centrifugation at 10,000 × g for 20 minutes at 4°C to remove insoluble material and aggregated proteins. The clarified supernatants were prepared for Western blot analysis by adding 4× Sample Buffer (Thermo Fisher Scientific, NP0008) and boiling.

Surface Plasmon Resonance (SPR) assay

Surface Plasmon Resonance (SPR) analysis was performed by MedChemExpress to investigate the interaction between Human FAM3C protein and Tucatinib. The Human FAM3C protein (MedChemExpress, HY-P70885) was immobilized onto a CM5 chip using amine coupling. The protein coupling buffer was 1.0×PBS-P+ (pH 7.4), and the interaction buffer was 1.0×PBS-P+ (pH 7.4) supplemented with 5% (v/v) DMSO.

Briefly, for protein immobilization, Channel 4 of a CM5 sensor chip was activated with 1-ethyl-3-(3-dimethylaminopropyl) carbodiimide (EDC, Cytiva) and N-hydroxysuccinimide

(NHS, Cytiva) at a flow rate of 10 μL/min. Human FAM3C protein, diluted to 50 μg/mL in sodium acetate buffer, was then immobilized onto Channel 4 at 10 μL/min. The surface was subsequently blocked with ethanolamine at 10 μL/min. Channel 3 served as a reference, undergoing the same activation and blocking steps but with immobilization buffer lacking protein.

For interaction testing, Tucatinib (MedChemExpress, 937263-43-9) was serially diluted across a range of concentrations in 5% DMSO-containing interaction buffer (Supplementary Table 7). The diluted Tucatinib samples were then injected over the immobilized FAM3C protein (from lowest to highest concentration) at a flow rate of 30 μL/min for 150 seconds. After each concentration injection, the chip surface was regenerated using 10 mM glycine-HCl (pH 2.0) solution for 5 minutes. Binding and dissociation constants were determined by globally fitting the acquired sensorgram data to a 1:1 Langmuir binding model using Biacore Insight Evaluation Software (Cytiva, Marlborough, MA, USA).

Code availability

No custom code was generated for this study.

Supplemental References

1. Suvà ML, Rheinbay E, Gillespie SM, Patel AP, Wakimoto H, Rabkin SD, et al. Reconstructing and reprogramming the tumor-propagating potential of glioblastoma stem-like cells. *Cell*. 2014;157(3):580-94.
2. Mack SC, Singh I, Wang X, Hirsch R, Wu Q, Villagomez R, et al. Chromatin landscapes reveal developmentally encoded transcriptional states that define human glioblastoma. *J Exp Med*. 2019;216(5):1071-90.
3. Qiu Z, Zhao L, Shen JZ, Liang Z, Wu Q, Yang K, et al. Transcription Elongation Machinery Is a Druggable Dependency and Potentiates Immunotherapy in Glioblastoma Stem Cells. *Cancer Discov*. 2022;12(2):502-21.
4. Castellan M, Guarnieri A, Fujimura A, Zanconato F, Battilana G, Panciera T, et al. Single-cell analyses reveal YAP/TAZ as regulators of stemness and cell plasticity in Glioblastoma. *Nat Cancer*. 2021;2(2):174-88.
5. Yuan H, Wu X, Wu Q, Chatoff A, Megill E, Gao J, et al. Lysine catabolism reprograms tumour immunity through histone crotonylation. *Nature*. 2023;617(7962):818-26.
6. Jafari R, Almqvist H, Axelsson H, Ignatushchenko M, Lundbäck T, Nordlund P, et al. The cellular thermal shift assay for evaluating drug target interactions in cells. *Nat Protoc*. 2014;9(9):2100-22.
