# Supplementary material for: Cancer stem cells synthesize proline to attenuate oxidative stress
Source: J Clin Invest. 2026 Jun 1;136(11):e200775. doi: 10.1172/JCI200775 (PMC13221224; doi:10.1172/JCI200775)

**Full unedited blot/gel for Figure 1**

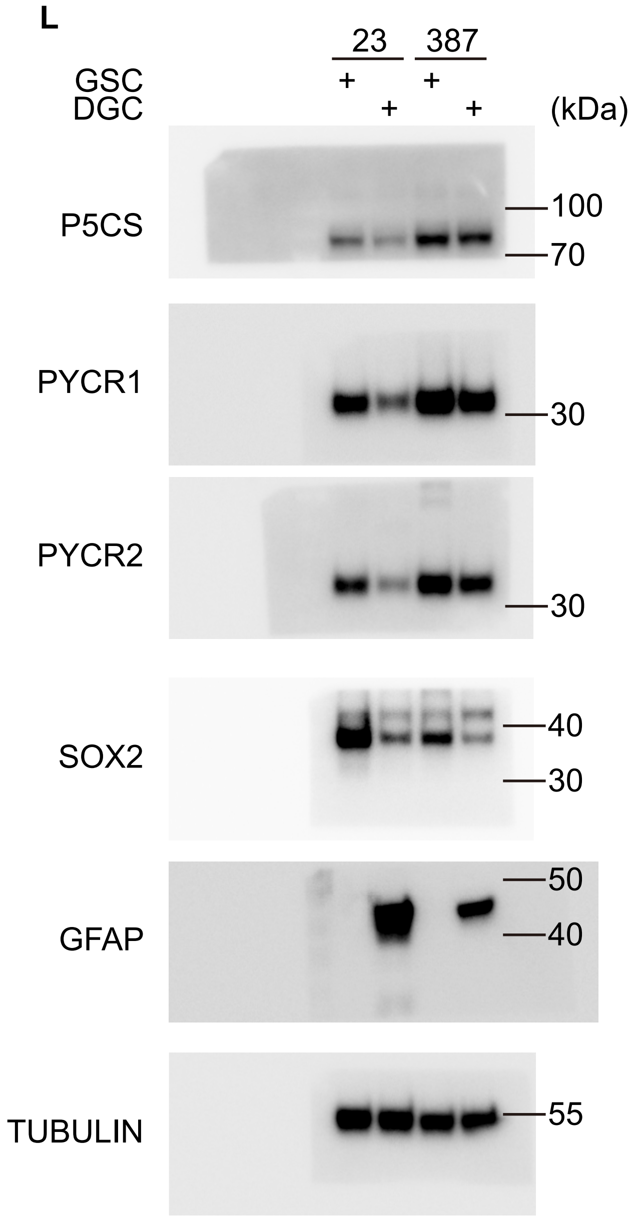

**Full unedited blot/gel for Figure 2**

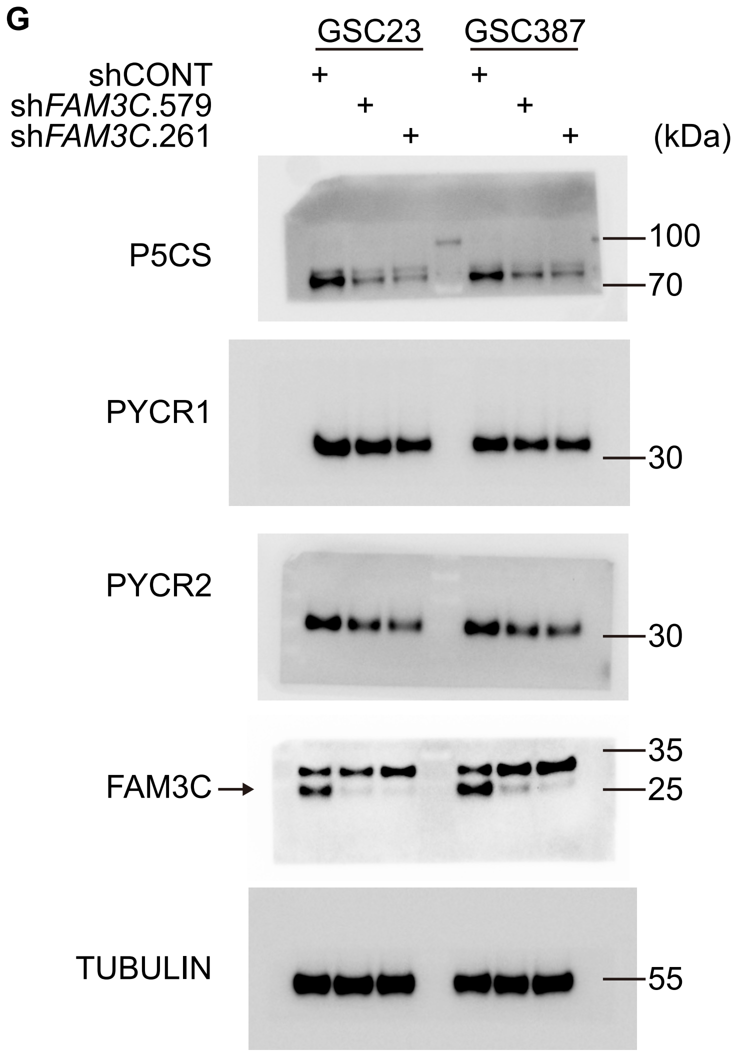

## Full unedited blot/gel for Figure 3

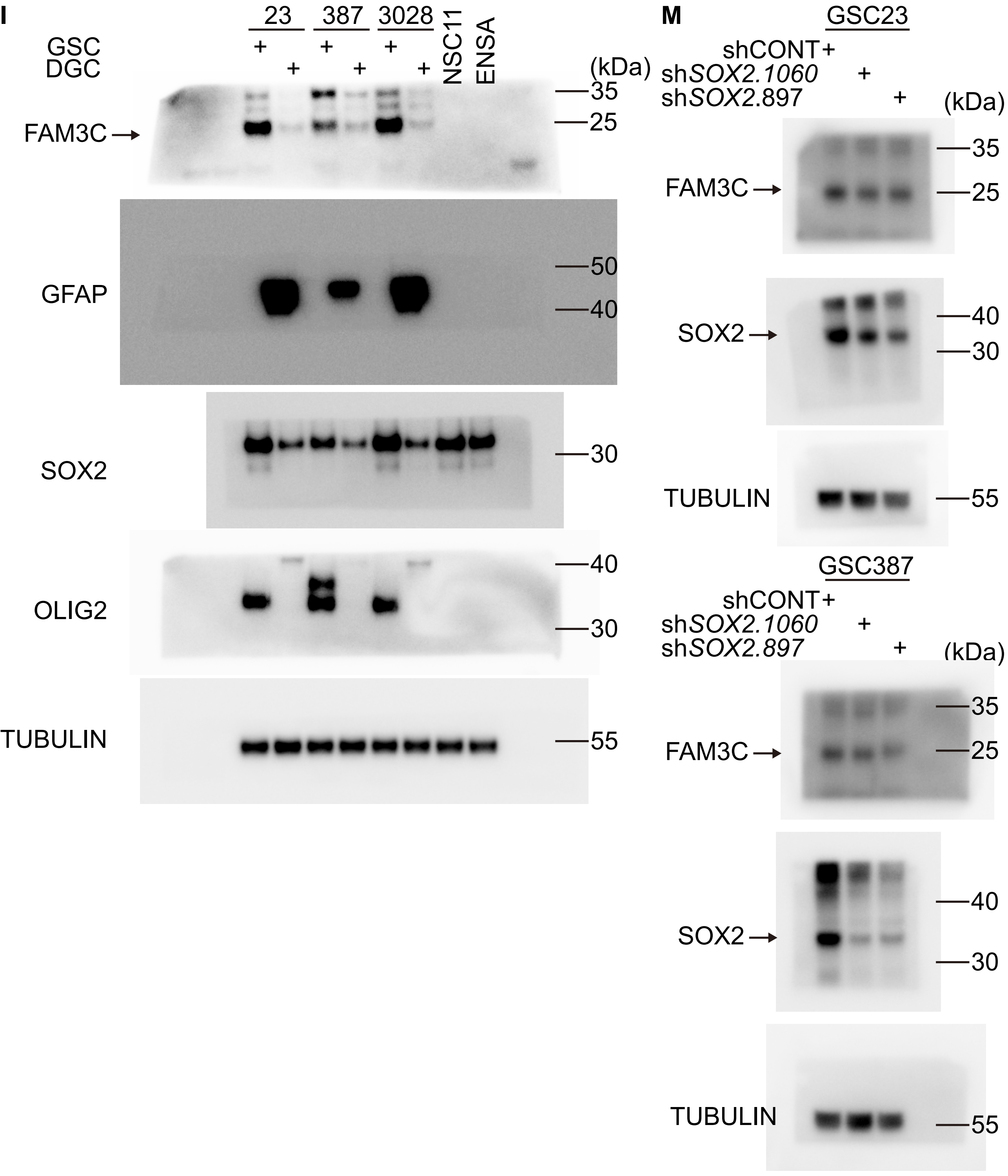

**Full unedited blot/gel for Figure 4 (continued on next page)**

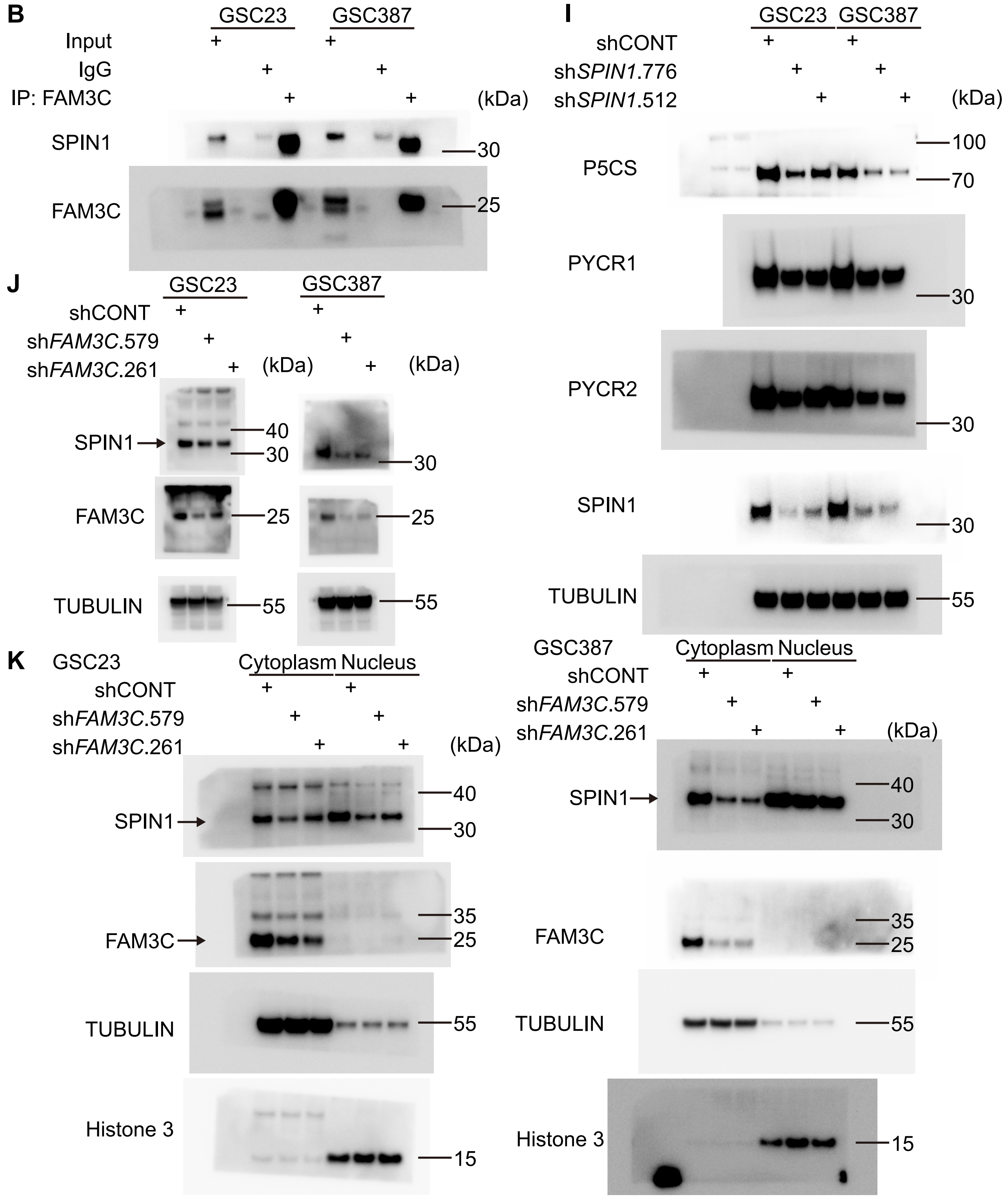

**Full unedited blot/gel for Figure 4 (continued)**

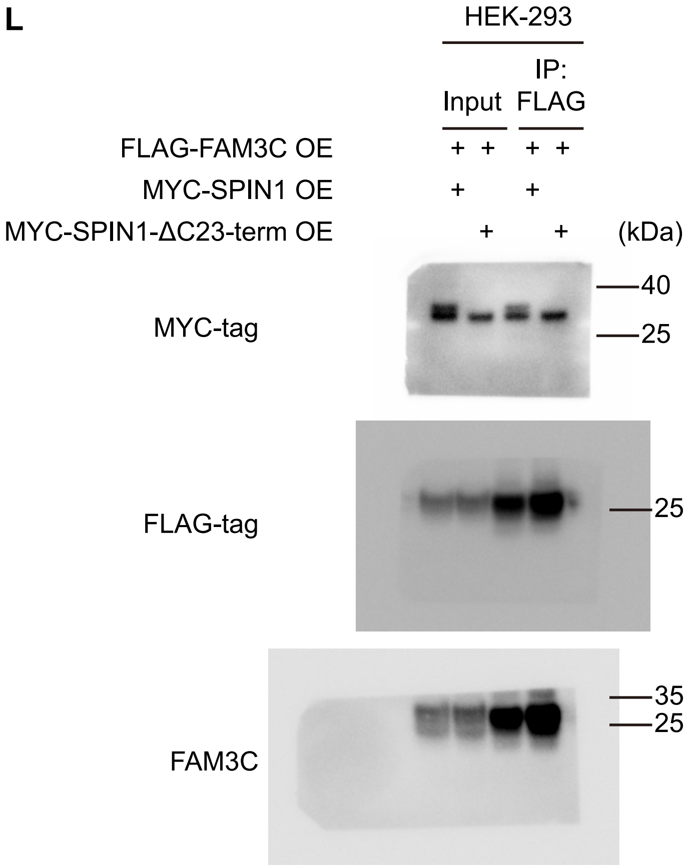

## Full unedited blot/gel for Figure 5

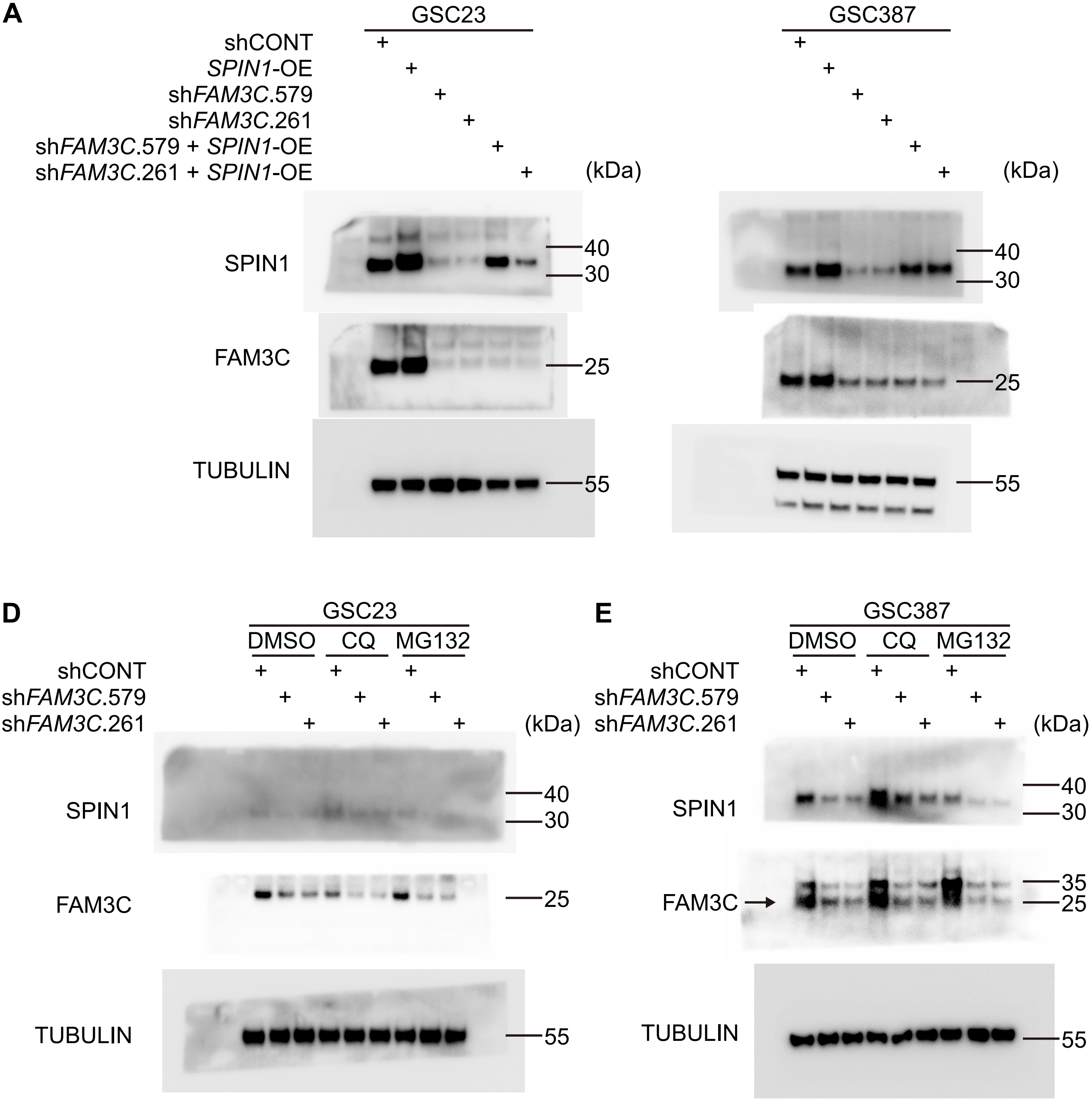


Note: CQ = Chloroquine.

## Full unedited blot/gel for Figure 7

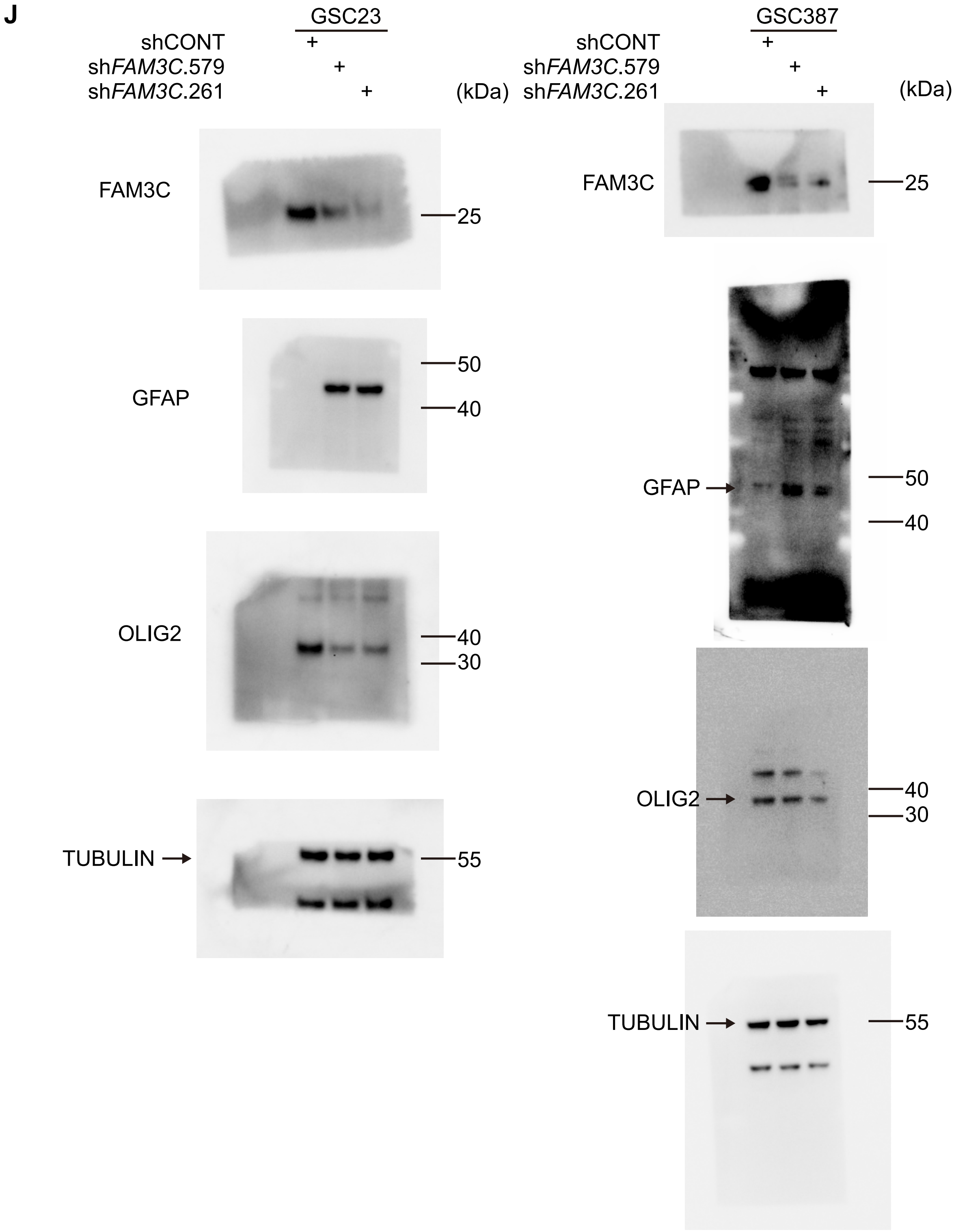

**Full unedited blot/gel for Figure 8**

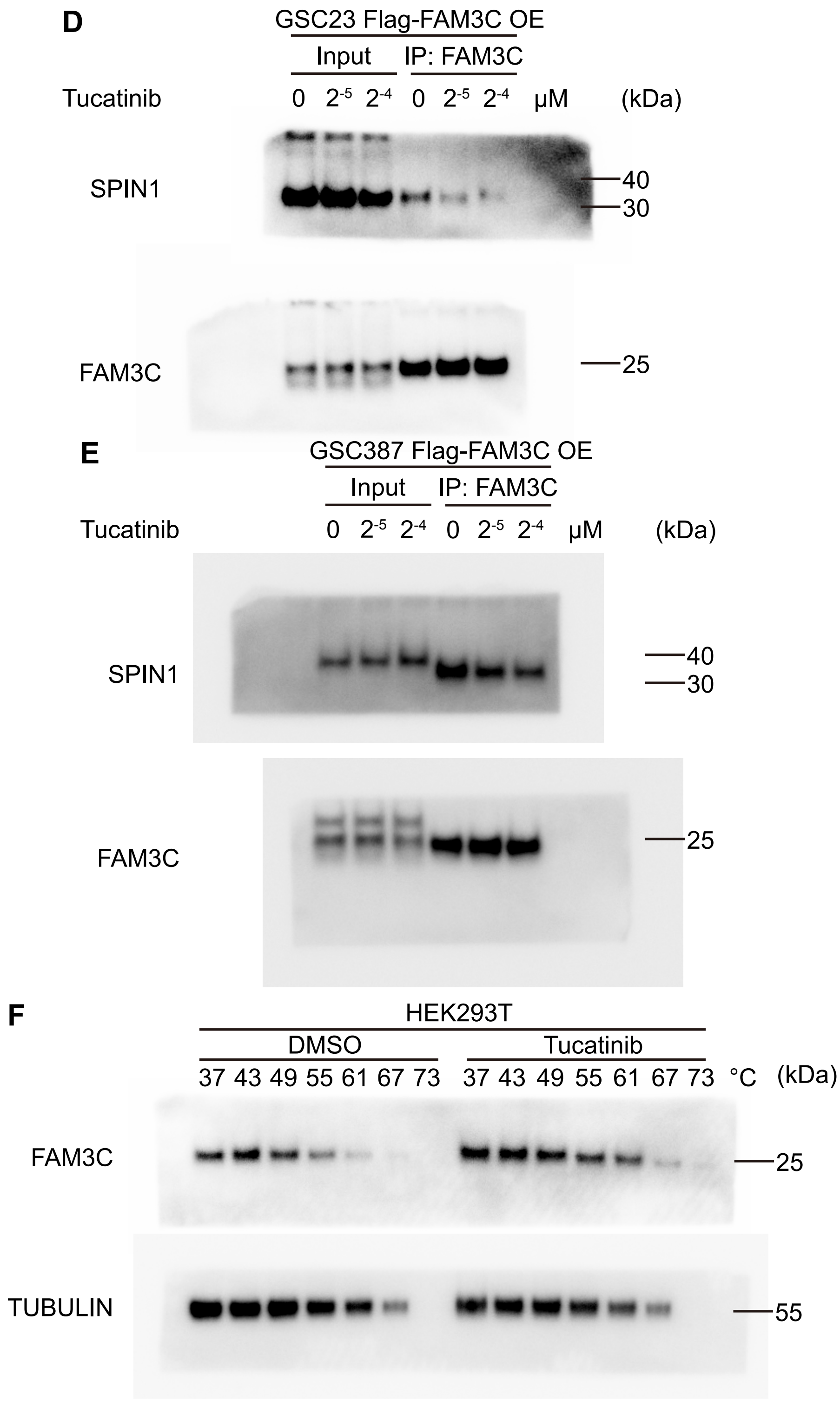

**Full unedited blot/gel for Supplemental Figure 1**

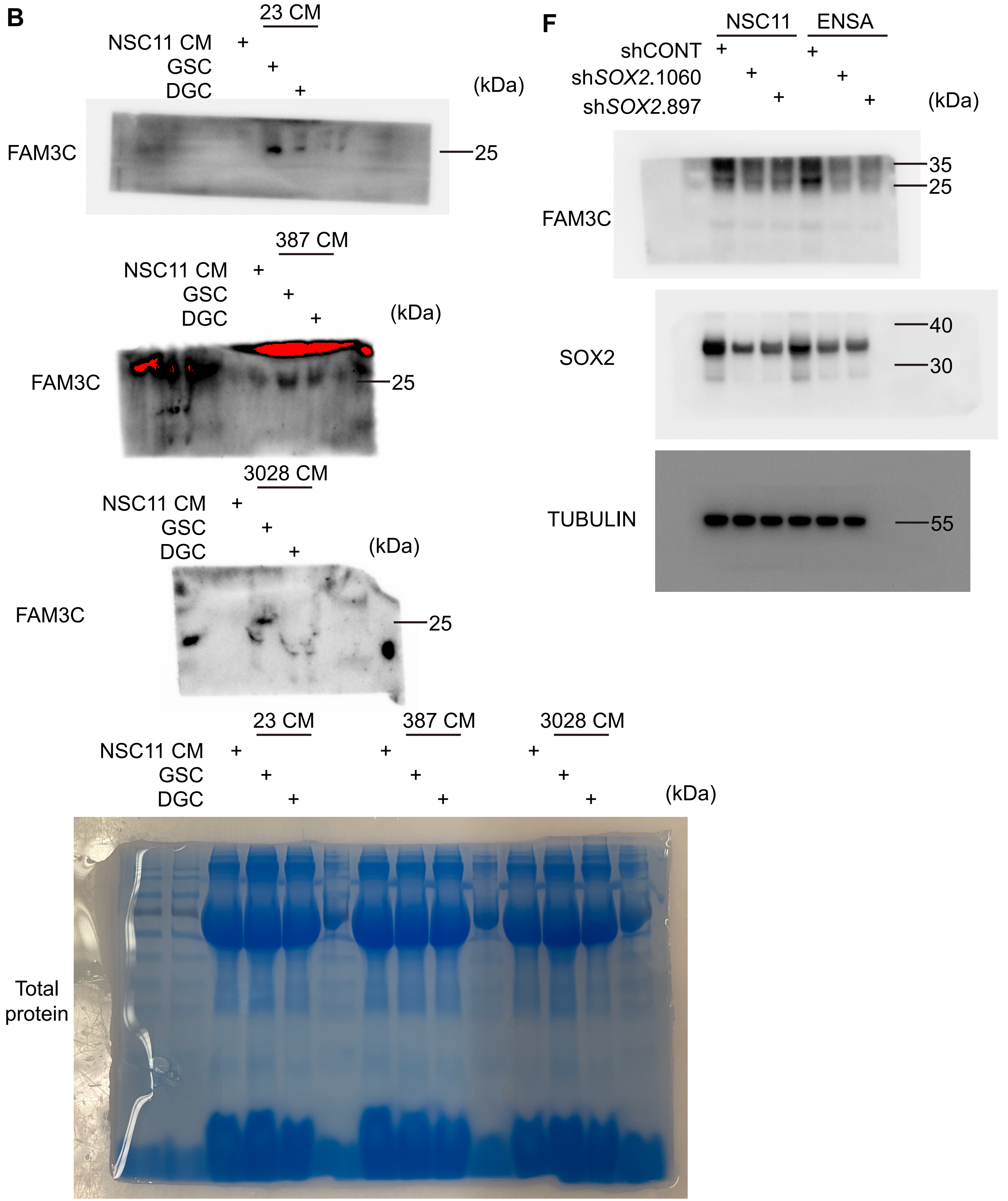

**Full unedited blot/gel for Supplemental Figure 2**

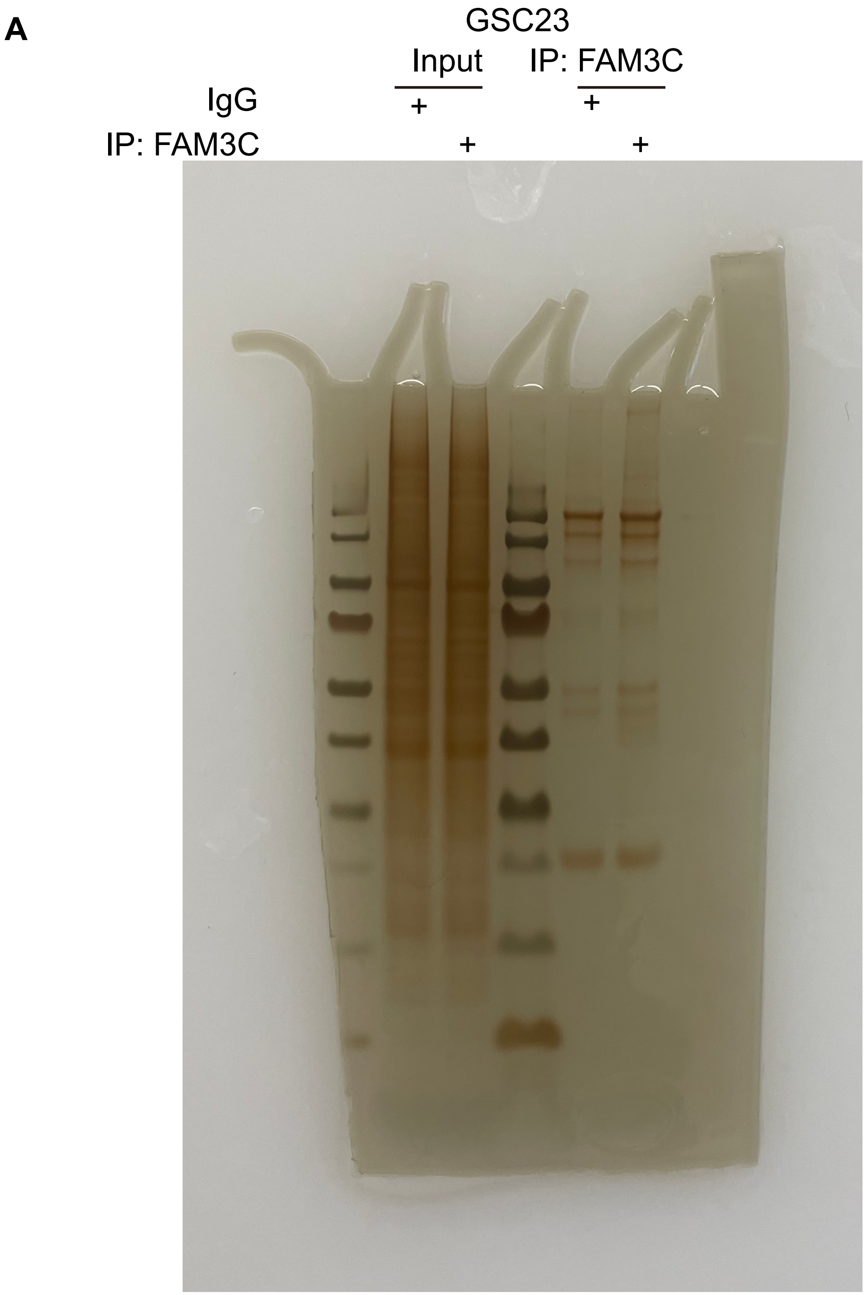

**Full unedited blot/gel for Supplemental Figure 7**

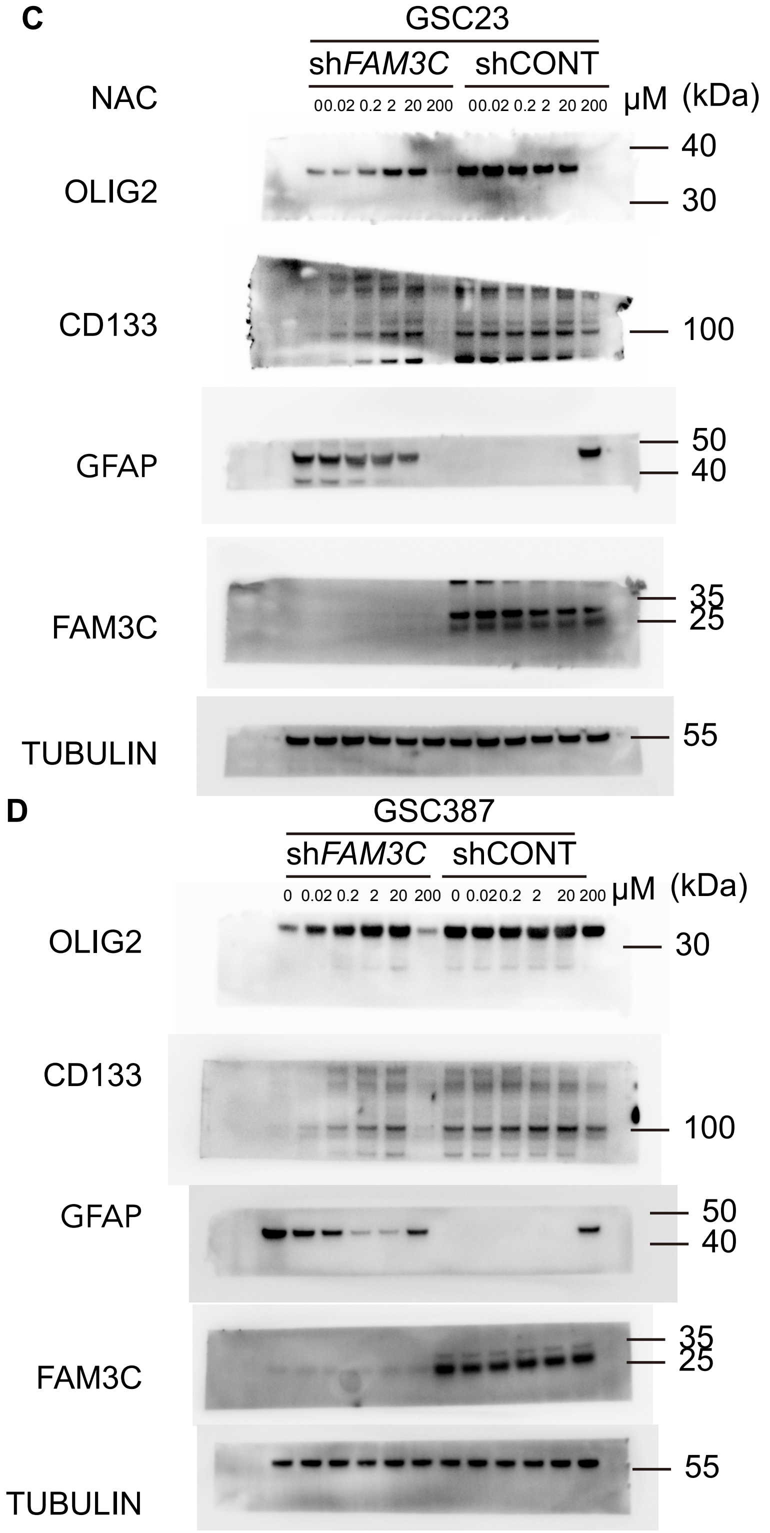

Supplement: Unedited blot and gel images [file jci-136-200775-s040.pdf]
